# The regulation of cell metabolism by hypoxia and hypercapnia

**DOI:** 10.1016/j.jbc.2025.108252

**Published:** 2025-02-04

**Authors:** Ben Reddan, Eoin P. Cummins

**Affiliations:** 1 School of Medicine, University College Dublin, Dublin, Ireland; 2 Conway Institute of Biomolecular and Biomedical Research, University College Dublin, Dublin, Ireland

**Keywords:** hypoxia, hypercapnia, metabolism, oxygen, carbon dioxide, mitochondria, tumor microenviroment, glycolysis, oxidative phosphorylation, lipid metabolism, amino acid metabolism

## Abstract

Every cell in the body is exposed to a certain level of CO_2_ and O_2_. Hypercapnia and hypoxia elicit stress signals to influence cellular metabolism and function. Both conditions exert profound yet distinct effects on metabolic pathways and mitochondrial dynamics, highlighting the need for cells to adapt to changes in the gaseous microenvironment. The interplay between hypercapnia and hypoxia signaling is the key for dictating cellular homeostasis as microenvironmental CO_2_ and O_2_ levels are inextricably linked. Hypercapnia, characterized by elevated pCO_2_, introduces metabolic adaptations within the aerobic metabolism pathways, affecting tricarboxylic acid cycle flux, lipid, and amino acid metabolism, oxidative phosphorylation and the electron transport chain. Hypoxia, defined by reduced oxygen availability, necessitates a shift from oxidative phosphorylation to anaerobic glycolysis to sustain ATP production, a process orchestrated by the stabilization of hypoxia-inducible factor-1α. Given that hypoxia and hypercapnia are present in both physiological and cancerous microenvironments, how might the coexistence of hypercapnia and hypoxia influence metabolic pathways and cellular function in physiological niches and the tumor microenvironment?

Molecular oxygen (O_2_) is the primary gaseous substrate of cellular metabolism while carbon dioxide (CO_2_) is the primary gaseous byproduct. Gas exchange occurs by diffusion during respiration, allowing O_2_ and CO_2_ to move between the alveoli and capillary blood. The efficiency of this gas exchange in the lungs is influenced by the rate of blood flow through the capillaries and the effectiveness of gas diffusion across the alveolar membrane (1). Upon diffusing across the alveolar membrane, hemoglobin in red blood cells enables O_2_ delivery to tissues and facilitates CO_2_ removal (2, 3, 4).

Disruptions in O_2_ or CO_2_ levels outside the physiological range pose significant risks to cellular, tissue, and overall organismal survival. Respiratory conditions such as chronic obstructive pulmonary disease (COPD) and acute respiratory distress syndrome severely impair lung function, disrupting the O_2_/CO_2_ diffusion balance. This disruption can lead to the development of hypoxia and/or hypercapnia systemically and in tissue microenvironments (5). Alterations in O_2_ and CO_2_ levels can significantly impair cellular metabolism and processes. Under normal conditions, many cells rely on aerobic respiration within mitochondria for efficient ATP production. However, in a hypoxic environment, cells shift to anaerobic glycolysis, a rapid process that generates ATP without O_2_ but produces fewer molecules of ATP, and results in the accumulation of lactate. On the other hand, elevated CO_2_ levels (hypercapnia) can alter the cellular metabolic profile (6), affect protein function, and dampen the inflammatory response of the innate immune system *e.g.* through reduced cytokine expression. Thus, the balance of O_2_ and CO_2_ is crucial for efficient energy production and normal physiological function. The cellular response to altered levels of O_2_ and CO_2_ represents an adaptive response to metabolic/energetic stress which will be discussed in the subsequent sections.

The following sections of this review will offer a foundational overview of the onset of hypercapnia and hypoxia in mammalian physiology, along with the biochemical components of cellular metabolic reactions, including key molecules, cofactors, and pathways involved in cellular metabolism. The review will then explore the effects of hypercapnia and hypoxia on these metabolic pathways, emphasizing how cells adapt to gaseous microenvironments to maintain function.

### Hypercapnia

CO_2_ is a fundamental byproduct of many metabolic reactions occurring within the body. Catabolism of one pyruvate molecule produces a total of three CO_2_ molecules, through three respective oxidative decarboxylation reactions—(i) the conversion of pyruvate to acetyl CoA as a precursor to tricarboxylic acid (TCA) cycle entry, (ii) the conversion of isocitrate to alpha-ketoglutarate (α-KG), and (iii) α-KG to succinyl CoA, which all occur within the mitochondria. Normal CO_2_ tension (*i.e.* normocapnia) in humans equates to a partial pressure of CO_2_ (pCO_2_) ranging from 35 to 45 mmHg. However, tissue CO_2_ levels may vary considerably depending on the physiological context. Hypercapnia is a term that describes the elevation of blood pCO_2_ above 45 mmHg, which may be a consequence of respiratory failure or changes in cellular metabolism (7). Elevated CO_2_ is also relevant in the context of cancer, as tumor microenvironments (TMEs) can be both hypoxic and hypercapnic. A hypercapnic microenvironment occurs due to the restriction of CO_2_ elimination and alterations in cellular metabolism, resulting in an increased production of CO_2_ (8) with pCO_2_ levels ranging from 59 to 84 mmHg in solid tumors (8, 9). Moreover, while the influence of hypoxia is more understood in the context of tumor metabolism, the metabolic implications associated with hypercapnia are less well understood.

Cancer cells generate substantial amounts of CO_2_, a byproduct of titrating acids (*e.g.*, lactate) with HCO_3_⁻, as well as from significant adaptations in mitochondrial-dependent enzymatic reactions (decarboxylation) and the pentose phosphate pathway (10). Although the cause of hypercapnia associated with respiratory failure differs from TME-induced hypercapnia, hypercapnia, and elevated CO_2_ consistently exert profound effects on cellular metabolism and mitochondrial function. Kikuchi, *et al.,* (8) reported that hypercapnia reduces mitochondrial oxidative phosphorylation (OXPHOS), oxygen consumption, and mitochondrial membrane potential. The authors also observed a decrease in reactive oxygen species (ROS) production, which appears to reduce the efficacy of anticancer agents.

Type 2 respiratory failure can occur when carbon dioxide is not effectively removed from the body. Notably, many studies examining hypercapnia have not investigated the influence of hypoxia and hypoxia-inducible factor-1α (HIF-1α) stabilization, resulting in findings that are exclusively attributed to elevated CO_2_ levels. It is important to note that TMEs often exhibit high levels of HIF-1α stabilization (11). With relevance to such TME models, hypercapnia has been shown to inhibit the activation of the HIF pathway, which may mediate tumor hypoxia-induced chemoresistance (12). The effects of hypercapnia on the HIF pathway are further discussed later in this review. In hypercapnic models that do not intersect with hypoxic microenvironments or HIF-1α stabilization, hypercapnia may drive distinct cellular adaptations.

Equilibrium of O_2_ and CO_2_ levels are continually regulated by central and peripheral chemoreceptors which detect rapid fluctuations of O_2_ and CO_2_ through neural mechanisms *e.g.* pH sensitive ion channels, which is essential to maintain homeostasis (13). Although mammalian chemosensing mechanisms have been extensively investigated, the transcriptional responses associated with elevated CO_2_ levels beyond normal physiological ranges remain less understood. Research to date has explored the effects of chronic hypercapnia, permissive hypercapnia (a consequence of clinical protective ventilation strategies), and therapeutic hypercapnia where pCO_2_ levels are deliberately manipulated for clinical benefit. Clinical studies have observed that hypercapnic patients, including those with COPD, experience an increased 30-day mortality and a greater need for intensive care unit care (14). Additionally, *in vitro* studies have shown that hypercapnia can accelerate muscle atrophy (15) and induce metabolic reprogramming in immune cells (6). Therapeutic hypercapnia on the other hand, can be employed in clinical settings to assist in surgical procedures. In some instances, therapeutic hypercapnia can alter tissue pH, leading to hypercapnic acidosis, which in turn, may alleviate several inflammatory responses and aid vasodilation of smooth muscle cells (16, 17).

While no direct hypercapnia-responsive elements or hypercapnia-inducible factors have been identified, CO_2_ can modulate cellular function through posttranslational modification (PTM) of proteins. Specifically, CO_2_ can react with neutral lysine ε-amino groups and N-terminal α-amino groups, modifying proteins such as RuBisCO (18), OXA-48 (a class D β-lactamase) and the PII metabolic signaling protein (19), as well as mammalian hemoglobin (20). These CO_2_-induced PTMs enable cells to dynamically respond to changes in pCO_2_ (21). Upon exposing HEK-293 cells to 5% CO_2_ or 10% CO_2_ ± tumor necrosis factor, the Cann group demonstrated that physiologically relevant CO_2_ levels (10% CO_2_) reduce ubiquitin conjugation by modifying a CO_2_-reactive lysine at position 48 (*K*_*48*_), thereby inhibiting ubiquitin-IκBα conjugation and subsequently preventing NF-κB translocation and nuclear transcription (22). Given the widespread role of ubiquitin in diverse biological processes, this CO_2_-dependent PTM has the potential to modulate cellular signaling on a broad scale. Using an orthogonal approach to the Cann group, King *et al.* 2022 identified 20 CO_2_-reactive protein candidates, including the Calvin cycle enzyme fructose 1,6-bisphosphatase and the metabolic sensor protein PII. The authors proposed that the CO_2_-reactive lysine (*K*_*90*_) in PII acts as a negative regulator by antagonizing ATP binding, thereby inhibiting ATP hydrolysis in both *Synechocystis* sp. (Cyanobacteria) and *Arabidopsis thaliana* (thale cress). This hypothesis was validated using a BODIPY-FL-ATP-γ-S binding assay within a KOCN (isocyanate)/CO_2_ competition model, demonstrating the importance of *K*_*90*_ and that the CO_2_-dependent modification of PII can indeed reduce ATP binding. These findings highlight a potential mechanism by which CO_2_ may modulate protein function through reversible lysine modifications, influencing cellular responses to fluctuating CO_2_ levels. Thus, both the Cann and King approaches suggest that there are multiple CO_2_-reactive lysine residues in the mammalian proteome. Despite this, there are currently few experimentally validated mammalian proteins with verified CO_2_-dependent modifications (hemoglobin, ubiquitin and connexin 26) (23). It is likely that this number will increase with improvements in mass spectrometry protocols and efforts to identify modifiable N-terminal residues, which to date have not been targeted in their approaches. Developments in this area will provide new insights into how CO_2_ modulates protein networks and cellular responses, with potential implications for understanding diseases linked to CO_2_ dysregulation.

### Hypoxia

OXPHOS is reliant on O_2_ availability for sufficient ATP synthesis *via* the TCA cycle and electron transport chain (ETC). The requirement for and use of O_2_ for ATP production is referred to as aerobic metabolism. When oxygen availability becomes limited, and O_2_ demand exceeds supply, the cellular microenvironment becomes hypoxic. Hypoxia is a well-established hallmark of various pathological conditions. Hypoxia can develop when the balance between CO_2_ and O_2_ exchange is disrupted due to impaired alveolar function (*e.g.* lung disease) or from insufficient oxygen delivery to tissues. (*e.g.* stroke, myocardial infarction, cancer, and obstructive sleep apnea) (24).

HIFs are oxygen-sensitive transcription factors. Hypoxia and HIFs are well-documented for their influential role in the metabolic reprogramming of cell metabolism and ATP production (25). A detailed review of the HIF pathway is beyond the scope of this review but has been expertly reviewed elsewhere (26, 27, 28). In simple terms, hypoxia and HIFs induce metabolic adaptions that reduce the cell's dependence on oxygen-dependent energy production. Instead, cells become more dependent on anaerobic glycolysis, which does not require oxygen, to produce ATP during low-oxygen conditions (29, 30).

HIF-prolyl hydroxylases (PHDs) use oxygen, α-KG, ascorbate, and Fe^2^⁺ to hydroxylate HIF-1α, leading to its degradation *via* Von Hippel Lindau protein (VHL). Factor-inhibiting HIF also regulates HIF-1α through oxygen-dependent asparagine hydroxylation. In hypoxia, reduced hydroxylation by PHDs and factor-inhibiting HIF stabilizes and activates HIF-1α, which then dimerizes with HIF-1β. The HIF-1α/β dimer binds to hypoxic response elements within the promoters of hypoxia-responsive genes, initiating transcriptional activation (31). In addition, hypoxia and hypoxic related adaptations may be achieved pharmacologically *via* inhibition of PHDs through the administration of pharmacological HIF modulators *e.g.* roxadustat (FG-4592), an α-KG mimetic (24). Moreover, metabolic dysregulation *e.g.* changes in succinate and fumarate can also contribute to the stabilization of HIF-1α (32). This stabilization creates a pseudohypoxic environment, which promotes anaerobic glycolysis, increases erythropoiesis, and reduces aerobic respiration.

## Metabolism

Cellular metabolism consists of a series of complex, multienzyme reactions that provide cells with the biochemical foundation needed to maintain homeostasis. This homeostatic state is sustained by generating chemical energy through reactions that transfer carbons, protons, and electrons between molecules. A common metabolic reaction is the hydrolysis of ATP to ADP, releasing inorganic phosphate (P_i_) and energy (7.3 kcal/mol, or 30.5 kJ/mol) (33, 34). ATP synthesis is the end product of anaerobic and aerobic metabolism of glucose, lipids, and amino acids which is achieved by glycolysis, the TCA cycle and the ETC (33). Importantly, cellular metabolism entails much more than just energy provision, for example, generating essential biomolecules required for cellular homeostasis such as *de novo* lipogenesis (DNL) which converts metabolic intermediates into cellular lipids (*i.e.* phospholipids and triglycerides). These lipid molecules are indispensable for the maintenance of cellular homeostasis, proliferation, and structural integrity of cellular membranes (35).

### Glycolysis

Glycolysis is a metabolic pathway which catabolizes glucose to rapidly produce energy for cellular functions. The glycolytic pathway consists of 10 enzymes and is the initial point of entry for glucose utilization in cellular respiration. The glycolytic pathway can operate in both the presence or absence of O_2_. Glycolytic metabolism occurs within the cytosol in a two-phase process—the first being the “preparatory phase” hydrolyzing two ATP molecules, and the second being the “payoff phase” synthesizing ATP, pyruvate, and NADH (36).

When sufficient O_2_ is present, most cells will shuttle pyruvate to the TCA cycle for aerobic respiration, while in hypoxia, many cells upregulate glycolytic pathway activity. Many cancer cells preferentially use aerobic glycolysis, a phenomenon known as “Warburg metabolism” (37). The glycolytic product pyruvate can either enter the TCA cycle to undergo OXPHOS, be converted into alanine by the alanine amino transferase, or remain within the cytoplasm to be converted to lactate (38).

### Mitochondrial respiration

Mitochondria are double-membraned organelles which play dynamic roles in an array of cellular processes such ATP production, biosynthesis, and signaling. They regulate cellular functions through organelle-to-organelle communication, such as nuclear-mitochondrial cross-talk facilitated by factors such as mitochondrial transcription factor A. Additionally, mitochondria act as sensors of metabolic, biochemical, and local or systemic cues, enabling the mitochondria to modulate organismal processes in response to diverse stimuli (39). Metabolic signaling mechanisms, such as AMP-activated protein kinase (AMPK), play a critical role by regulating mitochondrial metabolism (40). This can be achieved by inhibition of anabolic processes resulting in the suppression of ATP hydrolysis *e.g.* AMPK-dependent suppression of mTORC1 (41), stimulating catabolic processes *e.g.* AMPK upregulation of PFK1, a rate limiting enzyme in the glycolytic pathway (40) and AMPK-dependent regulation of PGC-1α expression (42). A comprehensive review of AMPK function has been published previously (40). Thus, mitochondria and their metabolites are key regulators of metabolism.

### Tricarboxylic acid cycle

The TCA cycle, also known as the Krebs cycle or citric acid cycle, is a crucial series of enzymatic reactions that occur in the mitochondrial matrix serving as a metabolic engine for ATP production and reducing equivalents while also providing pivotal signaling mechanisms for cellular immunity (39, 43). During aerobic metabolism, the TCA cycle facilitates the removal of carbon molecules from metabolites (decarboxylation) (44). Per one molecule of acetyl-CoA that enters the TCA cycle, 2 CO_2_ molecules are produced by the oxidative decarboxylation of isocitrate and α-KG (45). The dynamics of the TCA cycle has been reviewed expertly elsewhere (39, 43).

### Electron transport chain

The ETC is comprised of five protein complexes which generate and maintain an electrochemical gradient between the mitochondrial inner membrane space and the mitochondrial matrix–the state of this gradient is referred to as mitochondrial membrane potential (ΔΨm). The main purpose of the ETC is to carry out OXPHOSto generate ATP by cleaving electrons (e^-^) and protons (H^+^) from TCA cycle products (NADH, succinate and so on). The dynamics and adaptations of the ETC has been expertly reviewed elsewhere (43, 46, 47, 48, 49, 50, 51).

Similar to the TCA cycle, the ETC is not a unidirectional electron pump. The ETC can accommodate reverse electron flow through complex I to reduce NAD^+^ to NADH. Reverse electron transfer can occur when the Q pool is highly reduced (excessive CoQH_2_) and ΔΨm is high due to a large flow of H^+^ ions out of the mitochondrial matrix. As a result, this shift in the NAD^+^/NADH balance leads to a substantial generation of ROS (52), influencing macrophage responses and metabolic dynamics (48).

### Lipid metabolism

The oxidation of fatty acids to acetyl-CoA is an extremely high energy-yielding process which occurs alongside glucose and amino acid metabolism. The theoretical values suggest that the complete oxidation of a palmitate (16-carbon lipid) molecule provides a maximum yield of 108 ATP molecules (53), while a single glucose molecule only provides a maximum yield of 32 ATP molecules (33). However, the respective processes and pathways which catabolize glucose, fatty acids, and amino acids to substrates for the TCA and ETC vary considerably.

Acyl-CoA synthetase (ACS) converts free fatty acids into fatty acyl-CoA, which are then transported into the mitochondria *via* the carnitine shuttle, forming fatty acylcarnitine. Following the translocation of fatty acids into the mitochondria, fatty acids become oxidized by a process called β-oxidation. An entire cycle of β-oxidation results in the formation of one acetyl-CoA molecule, NADH, FADH_2_, and electrons (54, 55).

### Amino acid metabolism

In the context of cellular metabolism, particularly central carbon metabolism, catabolism of amino acids requires the cleavage of the α-amino group from the carbon skeleton, enabling nutrient utilization. Glutamine, a nonessential amino acid and a preferred fuel for rapidly dividing cells, including enterocytes, lymphocytes, macrophages, and tumors (56), is translocated to the mitochondria through solute carrier transporter proteins (57). Glutaminase (GLS) activity then catalyzes the oxidative deamination of glutamine by removing of the α-amino group of glutamine, leading to the formation of glutamate (58, 59). Mitochondrial glutamate may be converted by glutamate dehydrogenase to form α-KG. This process integrates products of glutamine metabolism into the TCA cycle, facilitating their oxidation to succinyl Co-A or alternatively, engaging in reductive carboxylation to generate isocitrate (60, 61). This clearly demonstrates the link between amino acid metabolism and the TCA cycle.

The remaining amino acids play a crucial role in cellular function by serving as metabolic precursors for various biomolecules, including nucleotide bases and cellular signaling molecules. For instance, cysteine, glycine, and glutamate contribute to the synthesis of glutathione, a key cellular antioxidant, while tryptophan is a precursor for the neurotransmitter serotonin (62).

## Hypercapnia and metabolism

There is a growing interest in the influence of elevated CO_2_ on metabolism due to the importance of CO_2_ physiologically and the clinical burden of hypercapnia in patients. Moreover, there has been considerable attention directed toward the impact of hypercapnia on acid-base homeostasis and the development of hypercapnic acidosis. This review will concentrate on the CO_2_-dependent adaptations rather than those associated with (hypercapnic) acidosis. Despite the growing body of research on hypercapnia, detailed metabolism orientated studies remain relatively scarce. Research to date demonstrates that hypercapnia may be responsible for cellular metabolic reprogramming and altering gene expression related to aerobic and anaerobic metabolism.

### Hypercapnia and glycolysis

Research directly examining the impact of hypercapnia on glycolysis is limited, but some studies have examined the effect of hypercapnia on factors related to the glycolytic pathway. Phelan *et al.* conducted a study investigating the effects of 4-h exposure to elevated CO_2_ (10%) under pH-buffered conditions on gene expression in THP-1 monocytes and in an IL4-polarized bone-marrow-derived macrophage model. A cohort of genes was reported to be significantly affected, including a decreased *mRNA* expression of the glycolytic enzyme aldolase A (*ALDOA*). These findings were consistent in both immune cell models.

Aldolase, an enzyme from the “preparatory phase” of glycolysis and holds additional “moonlighting functions” in cellular metabolism beyond its role in glucose metabolism and glycolysis (63). During states of glucose deprivation, specifically when fructose-1,6-bisphosphate concentrations are low, aldolase assumes a role in the promotion of AMPK phosphorylation to upregulate alternative metabolic pathways such as OXPHOS, β-oxidation, or glutaminolysis (64, 65).

A separate study demonstrated that hypercapnia can suppress HIF signaling. These findings may support the hypothesis proposed by Phelan, *et al.,* that hypercapnia exerts suppressive effects on the glycolytic pathway. This may be achievable through the hypercapnic-dependent suppression of HIF-1α (12). Selfridge *et al.*, reported that 4 h exposure to hypercapnia (10% CO_2_) led to reduced protein stabilization and transcriptional activity of chemically induced HIF-1α and HIF-2α across diverse cell lines and mouse models. Crucially, metabolic reprogramming in hypoxia is achieved by the HIF-1α dependent upregulation of genes to regulate the expression and activity of glucose transporters and glycolytic enzymes (66). Additionally, to compliment this shift in cellular metabolism, HIF-1α can further suppress OXPHOS (67). Activation of HIF-1α prevents the shuttling of pyruvate into the mitochondria *via* pyruvate dehydrogenase kinase 1 (PDK1)-dependent phosphorylation of PDH (67). As a consequence of pyruvate deprivation in the mitochondria, TCA cycle and ETC activity notably decreases. HIF-1α further enhances lactate dehydrogenase (LDH) expression increasing the rate of conversion of pyruvate to lactate, promoting the regeneration of NAD^+^ (68).

Thus, both of the hypercapnia studies mentioned above, identified elevated CO_2_ (10% CO_2_) as a potential suppressor of glycolytic metabolism, which could possibly be achieved through the hypercapnic suppression of HIF-1α. Given that hypoxia and hypercapnia coexist in different tissue microenvironments, the balance between hypoxia-dependent promotion of glycolysis and hypercapnia-dependent attenuation of glycolysis may determine the overall metabolic state.

### Hypercapnia, mitochondrial function, the TCA cycle, and the electron transport chain

Given what is currently known about the impact of hypercapnia on glycolytic enzyme and transcript expression (discussed above), it is reasonable to suggest that hypercapnia could further prompt metabolic reprogramming.

In mammalian cells, three isocitrate dehydrogenase (IDH) isoforms exist, each with respective coenzyme dependencies. IDH1 which is primarily located in the cytoplasm, while two isoforms IDH2 and IDH3 reside within the mitochondria. IDH1 and IDH2 are NADP^+^-dependent, catalyzing the conversion of isocitrate to α-KG while reducing NADP^+^ to NADPH. IDH3 is NAD^+^-dependent, facilitating the reduction of NAD^+^ to NADH for subsequent incorporation into the ETC. The first CO_2_ molecule produced in the TCA cycle is attributable to IDH2 and IDH3 activity by converting isocitrate to α-KG. Previous research demonstrated that 72 h exposure to hypercapnia (pCO_2_ 120 mmHg, approx. 15% CO_2_) suppresses protein expression of IDH2 *via miR-183* regulatory activity, hindering cell proliferation rates in both N12 fibroblasts and A549 epithelial cancer cells (69). This finding is particularly significant because IDH2, along with IDH3, plays a crucial role in the oxidative decarboxylation of isocitrate to α-KG within the TCA cycle, a critical process for energy production and the synthesis of metabolic intermediates. Disruption of IDH2 activity due to hypercapnia can significantly impair the efficiency of the TCA cycle, potentially compromising overall cellular metabolism and function.

In addition, Vohwinkel *et al.* demonstrated that supplementation of α-KG appeared to exert a compensatory effect, by rescuing cell proliferation from the adverse exposure to hypercapnia. α-KG, aside from being derived from glucose, can also originate from glutamate *via* glutamate dehydrogenase. Glutamine-derived α-KG bypasses the requirement for either IDH2 or IDH3 for α-KG synthesis further highlighting the dynamic and flexible nature of cellular metabolism. Moreover, NADP^+^ dependent IDH1 and IDH2 exhibit bidirectional functions unlike IDH3. IDH2 can facilitate both oxidative decarboxylation and reductive carboxylation within the mitochondria of isocitrate and α-KG (70). Thus, there is potential for other IDH isoforms to compensate for loss of IDH2 function in hypercapnia as well as other mechanisms to replenish α-KG into the TCA cycle *e.g. via* glutaminolysis.

There is limited literature exploring the effects of hypercapnia on mitochondrial and ETC activity in mammalian cells. However, through *RNA*-sequencing analysis, Phelan *et al.*, discovered that 4-h exposure to pH buffered hypercapnia (10% CO_2_) significantly increased nine mitochondrial encoded genes in THP-1 monocytes (*MT-MD4, MT-ND4L, MT-ND5, MT-ND6, MT-CO1, MT-CO2, MT-CO3, MT-TL2,* and *MT-TY*). In the presence of hypercapnia and a 2-h lipopolysaccharide (LPS) treatment, the study highlighted 18 genes which were significantly increased (*MT-ND1, MT-MD4, MT-ND4L, MT-ND5, MT-ND6, MT-CO1, MT-CO2*, *MT-CO3*, *MT-ATP6*, *MT-ATP8*, *MT-TC*, *MT-TE*, *MT-TL1*, *MT-TL2*, *MT-TN*, *MT-TQ*, *MT-TV*, and *MT-TY*).

**Figure 1:**
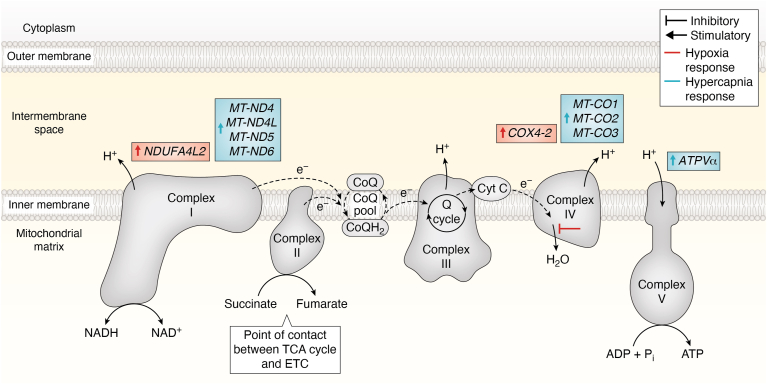
**Effect of hypoxia and hypercapnia on the electron transport chain (ETC).** This diagram illustrates the electron transport chain (ETC) within the inner mitochondrial membrane and its role in oxidative phosphorylation (OXPHOS). It depicts the sequential transfer of electrons through ETC complexes I-IV, which drives the pumping of protons across the membrane, creating a proton gradient. This gradient generates the mitochondrial membrane potential, which powers ATP synthesis *via* ATP synthase (complex V). The diagram highlights how the ETC converts the energy from electron transfer into ATP production. The figure also shows the effects of hypoxia and hypercapnia on these processes. *Red* stimulators and inhibitors represent the impact of hypoxia. *Blue* stimulators and inhibitors illustrate the effects of hypercapnia. ATPVα, ATP synthase subunit Vα; CoQ, coenzyme Q; COX4-2, cytochrome C oxidase subunit 4-2; ETC, electron transport chain; NDUFA4L2, NADH:ubiquinone oxidoreductase subunit A4-like 2; MT-ND, mitochondrial NADH dehydrogenase; MT-CO, mitochondrial cytochrome C oxidase; NADH/NAD^+^, nicotinamide adenine dinucleotide; P_i_, inorganic phosphate; TCA, tricarboxylic acid.

Complex I associated genes *MT-ND4, MT-ND4L, MT-ND5*, and *MT-ND6*, and complex IV associated genes *MT-CO1*, *MT-CO2*, *and MT-CO3*, exhibited CO_2_-dependent increases in transcript expression in both the absence and presence of LPS, indicating the robustness and consistency of these hypercapnic-sensitive changes. Furthermore, protein expression of mitochondrial membrane ATP synthase subunit 5α (ATP5α), was upregulated in response to hypercapnia. ATP5α serves as a catalytic subunit within ATP-synthase during OXPHOS (71) (Fig. 1).

Recent research additionally revealed that members of the orphan nuclear receptor family 4A (NR4A) are transcriptionally involved in the cellular response to buffered hypercapnia. Specifically, depletion of NR4A3 reduced the CO_2_ sensitivity of several mitochondrial protein-related genes *e.g.*, *MT-CO1* in THP-1 monocytes exposed to hypercapnia (10% CO_2_) for 4 h (Phelan *et al.* 2024). These findings suggest that NR4A3 transcriptional activity may influence mitochondrial function which impact TCA cycle activity, ETC activity, and ATP synthesis. To further elaborate on the hypercapnic effect on mitochondrial activity, the authors reported that there was no evidence of CO_2_-dependent changes on mitochondrial ROS production, but that 24-h exposure to hypercapnia significantly reduced mitochondrial cardiolipin levels, suggesting a possible link between CO_2_ and mitochondrial dysfunction. Additionally, the study revealed that 24-h hypercapnia exposure significantly increased cellular reductase activity as measured by [3-(4,5-dimethylthiazol-2-yl)-2,5-diphenyltetrazolium bromide] assay, suggesting alterations in various cytosolic subcompartments, including the mitochondria, in a THP-1 monocyte model. Overall, these results highlight the complex nature hypercapnia imposed on mitochondrial function and activity.

When considering the effect of hypercapnia on metabolism and metabolic regulators, a large body of research underscores the important role of AMPK and phospho-AMPK (pAMPK) expression in orchestrating metabolic rewiring to ensure adequate levels of ATP. This role for AMPK activation is primarily accomplished by enhancing other aerobic metabolic pathways, such as glutaminolysis and β-oxidation, in response to the suppression of glycolysis (72, 73). Moreover, hypercapnia has previously been demonstrated to influence pAMPK protein expression, contributing to the regulation of skeletal muscle phenotypes (74, 75). Differences in the metabolic profiles between type I (“slow twitch”) and type II (“fast twitch”) skeletal muscle phenotypes have been extensively documented elsewhere (76, 77) with type I skeletal muscle displaying highly oxidative characteristics with large mitochondrial abundance, while type II skeletal muscle displays a greater glycolytic capacity (75). Hypercapnia induced increases in type I skeletal muscle fiber abundance suggests that CO_2_ may be responsible for oxidative metabolic reprogramming of the extensor digitorum longus muscle of C57 mice exposed to hypercapnia (10% CO_2_) for 60 days (75). Notably, in the context of COPD skeletal muscle atrophy is induced (78). In hypercapnia, skeletal muscle myotube atrophy is driven by elevated AMPK, forkhead box O3 (FoxO3a) and muscle RING-finger protein-1 (MuRF1)-dependent proteolysis. Hypercapnic exposure has been linked to similar skeletal muscle atrophy mechanisms in C57 mice models (MuRF1 ^+^/^+^ and MuRF1^−/−^ litter mates). These mice were exposed to either 10% CO_2_ or room air for 3, 7, 14, and 21 days. The authors concluded that the presence of MuRF1 during hypercapnic exposure was responsible for reductions in grip strength and the cross-sectional area of the soleus muscle (15).

RNA-seq analysis has revealed that hypercapnia significantly alters the expression of genes involved in mitochondrial function, including those associated with ETC complexes, such as complex I, IV, and ATP synthase. These transcriptional changes are accompanied by corresponding alterations in ATP5α protein expression. In addition to these mitochondrial effects, Western blot analysis revealed that hypercapnia significantly impacts TCA cycle flux through *microRNA*-dependent suppression of IDH2. Supplementation with α-KG restores enzyme function, highlighting its critical role as a TCA cycle intermediate, and its potential as a therapeutic supplement for addressing metabolic disorders associated with hypercapnia. Additionally, hypercapnia affects protein expression of metabolic regulators like AMPK, leading to the reprogramming of aerobic metabolism, emphasizing the profound effects of elevated CO_2_ on cellular metabolism. While no CO_2_ specific transcription factor has been identified to date, it is evident that hypercapnia imposed a host of adaptations on the mitochondria.

Immune reactions are influenced by the crosstalk between signal transduction pathways and metabolic reprogramming events (79). For example, dysregulation of the TCA cycle intermediates and mitochondria can disrupt metabolic networks, causing the buildup of specific metabolites or danger molecules that activate inflammatory pathways—*e.g.* succinate-dependent stabilization of HIF-1α (80). Additionally, many intracellular innate immune receptors influence the metabolism and function of macrophages and T cells, underscoring their role in linking the innate immune system and metabolic function (81). For example, cyclic GMP-AMP synthase recognition of double-stranded DNA following *Brucella abortus* infection activates the STING pathway. STING activation subsequently repolarizes M2-like resolving macrophages into an M1-like inflammatory phenotype, which is associated with metabolic reprogramming. An impaired TCA cycle in these M1-like macrophages results in succinate accumulation. The elevated succinate levels inhibit PHD activity, leading to the stabilization of HIF-1α (81, 82, 83).

Hypercapnia disrupts several mitochondrial functions including the TCA cycle, potentially impeding anaplerotic reactions. As mitochondria decrease in size and the TCA cycle becomes dysregulated, the inability of hypercapnic cells to perform these critical anaplerotic reactions may lead to nutrient depletion or the preferential generation of certain metabolites over others. These disruptions have implications for the impact of hypercapnia on immunometabolism. While the exact mechanisms are not yet fully understood, these metabolic adaptations may interfere with the innate immune system and inflammatory signaling, supporting the theory that hypercapnia impedes immune function. While the concept of hypercapnia affecting immune function is not new, recent data detailing changes in immune cell metabolism underscore the need for further research into this area.

### Hypercapnia and lipid metabolism

Fatty acids serve as crucial metabolic substrates which fulfill diverse roles, essential for cell survival and proliferation. Cellular fatty acids can be acquired exogenously, through uptake across the cellular membrane, or synthesized endogenously *via* DNL synthesis. Once within the cell, cellular fatty acids may undergo catabolism *via* O_2_-dependent β-oxidation, which occurs predominantly within the mitochondria, or they may be also utilized for membrane reconstruction or the biosynthesis of cellular signaling molecules. In scenarios where glycolysis and glucose metabolism are downregulated such as low glucose availability or impaired glucose uptake *e.g.* diabetes mellitus, cellular energetics become more reliant on aerobic fatty acid metabolism (84).

Casalino-Matsuda *et al.* investigated the effects of 24-h exposure to hypercapnia (20% CO_2_) on primary normal human bronchial epithelial cells, cultured using a liquid-air interface (85). The authors observed that hypercapnia downregulated the expression of 183 genes and upregulated the expression of 126 genes. Interestingly, major gene clusters associated with lipid metabolism were found to be upregulated. Among these significantly upregulated genes were acyl-CoA dehydrogenases (*ACADs*), acyl-CoA synthetase short-chain family members (*ACSS*), fatty acid desaturase (*FADS*), and aldehyde dehydrogenases (*ALDH*), several of which are intimately associated with mitochondrial β-oxidation. Particularly noteworthy are the upregulation of *ACAD* genes, which process fatty acyl-CoA to acetyl CoA during β-oxidation to serve as a substrate for TCA cycle incorporation and *ACSS* genes, which are involved in the uptake and activation of short chain fatty acids to undergo metabolism (86). Moreover, increased expression of both fatty acid desaturase and *FAH* genes may suggest that hypercapnia induces fatty acid synthesis, while increased expression of aldehyde dehydrogenase genes may indicate an enhanced conversion of fatty aldehydes to fatty acids (87). Taken together, these demonstrate significant alterations in lipid metabolism pathways induced by hypercapnia in mammalian cells.

Interestingly, some of these previously reported findings were further corroborated by Phelan, *et al.* 2023 using RNA-sequencing. The authors demonstrated a significant upregulation of the *ACADS* gene in response to 24 h hypercapnia (10% CO_2_) exposure in both the absence and presence of LPS, using a THP-1 monocyte model. They also observed a significant increase in several acyl-CoA synthetase long-chain (*ACSL*) genes. While Casalino-Matsuda *et al.* (85) reported CO_2_ dependent changes primarily in short chain fatty acid associated genes, Phelan, *et al.* observed a more prevalent change in long-chain fatty acid associated genes. Thus, in different cellular models, using different degrees of CO_2_ exposure, both studies demonstrated the CO_2_-dependent effect on acyl-CoA synthase genes in response to hypercapnia and a robust hypercapnic dependent effect on *ACAD* and *ACS* gene expression. These data exemplify the effect of elevated CO_2_ on cellular fatty acid metabolism.

**Figure 2:**
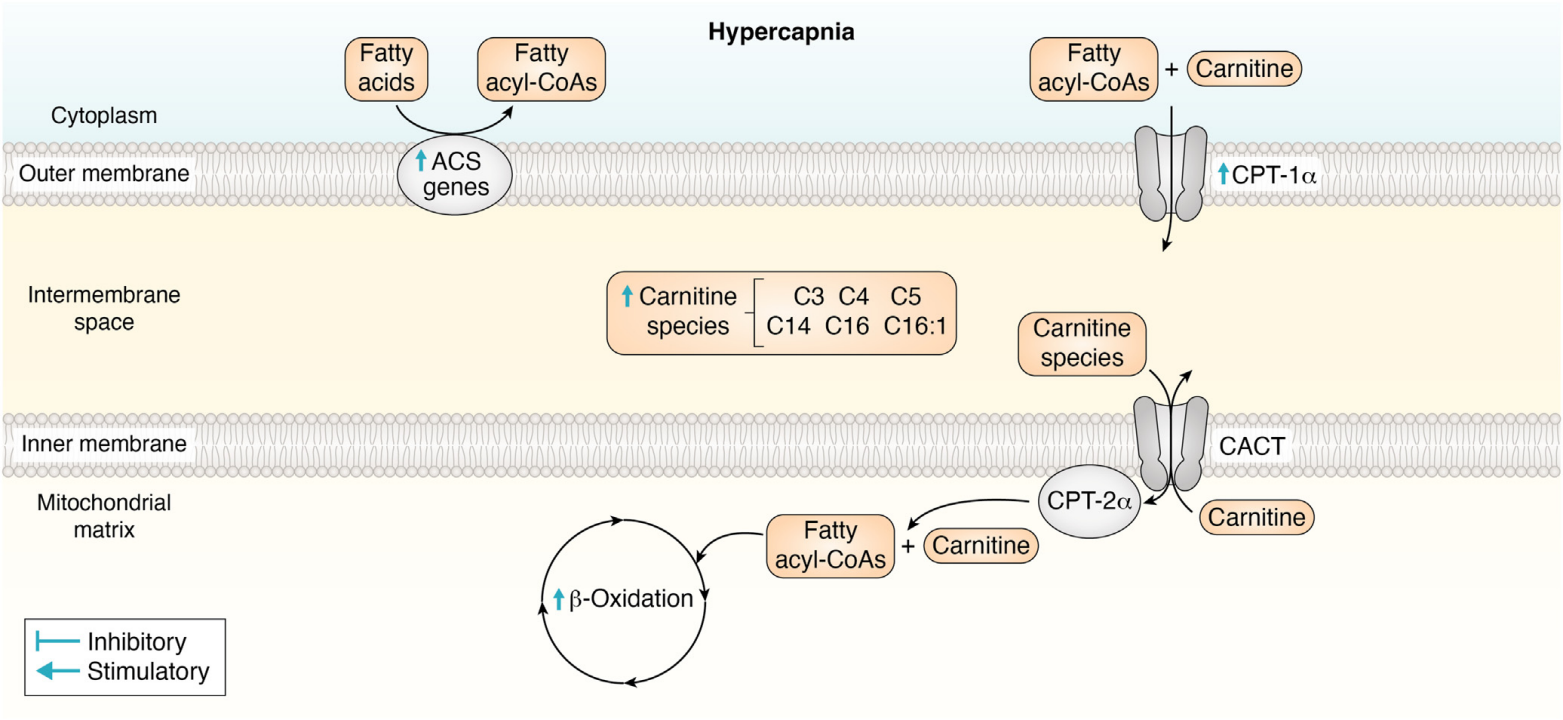
**Impact of hypercapnia on fatty acid uptake and incorporation into the mitochondrialβ-oxidation pathway.** The diagram highlights the impact of hypercapnia on the uptake and incorporation of intracellular fatty acids into the β-oxidation pathway. Fatty acids are shown entering the mitochondria *via* the carnitine shuttle, where they undergo β-oxidation to generate acetyl-CoA for the TCA cycle. Hypercapnia is depicted as influencing key steps in this pathway, potentially altering the efficiency of fatty acid transport and β-oxidation, thereby modulating mitochondrial energy metabolism under high CO₂ conditions. Blue stimulators and inhibitors illustrate the effects of hypercapnia. Incorporation refers to the uptake and integration of specific molecules or nutrients into cellular or metabolic structures. ACS, acyl-CoA synthase; CACT, carnitine acylcarnitine translocase; CPT-1α, carnitine palmitoyl transferase 1α; CPT-2α, carnitine palmitoyl transferase 2α; C3, propionylcarnitine; C4, butyrylcarnitine; C5, isovalerylcarnitine; C14, tetradecanoylcarnitine; C16, palmitoylcarnitine; C16:1, palmitoleylcarnitine; TCA, tricarboxylic acid.

Consistent with this concept, Phelan, D.E *et al.* employed LC-MS, to reveal that 4-h exposure to hypercapnia significantly altered the abundance of several acylcarnitine species (C3, C4, C5, C14, C16, and C16:1) within the mitochondria. These metabolites were significantly altered in both the absence and presence of LPS. As previously described, fatty-acyl CoA is transformed to acylcarnitine for the transport of fatty acids into mitochondria for subsequent β-oxidation. A process further facilitated by *ACSL* genes and by carnitine palmitoyl transferase 1α (CPT-1α) (72). Interestingly, the authors discovered that *CPT-1α* mRNA expression was also upregulated by hypercapnia in IL-4 polarized bone-marrow-derived macrophages after 24 h. CPT-1α is a rate-limiting enzyme which facilitates the transfer of acyl-CoA and carnitine, to acylcarnitine. This in turn, allows for the transport of fatty acid across the mitochondrial matrix for subsequent β-oxidation (Fig. 2).

Thus, independent studies conducted by both Casalino-Matsuda, *et al.* and Phelan *et al.* provide compelling evidence that hypercapnia impacts pathways associated with fatty acid synthesis and metabolism, as evidenced by changes in transcription and metabolite abundance. The full implications of CO_2_-dependent metabolic rewiring remain to be elucidated; however, a possible consequence is altered membrane permeability to CO_2_. Existing literature suggests that increases in cellular cholesterol content reduce CO_2_ permeability across the cell membrane (88) and a recent article reported that low levels of CO_2_ activate SREBP2 to induce cholesterologenic genes and total cell cholesterol (NIH3T3 cells exposed to 1% CO_2_ compared to 5% CO_2_ for 24 h) (89). Thus, we can speculate that altered lipid metabolism in hypercapnia may be an attempt to alter CO_2_ diffusion, and maintain environmental homeostasis, as well as provide additional substrates for aerobic metabolism; however, this hypothesis remains untested at present.

### Hypercapnia and amino acid metabolism

Amino acid metabolism is a pivotal pathway for energy provision and regular cellular function, with many amino acids undergoing synthesis or catabolism within the mitochondria. Amino acid restriction or depletion may influence an array of metabolic related processes such as TCA, respiratory chain activity, as well as mitochondrial biosynthesis. Additionally, dysfunctional amino acid synthesis and metabolism has been linked to primary mitochondrial diseases and diseases directly induced by mitochondrial dysregulation (90). For instance, increased circulating levels of alanine have been associated with disruptions in pyruvate synthesis and metabolism, often due to changes in the function of pyruvate-specific enzymes (91).

In addition to describing changes in acylcarnitine abundance in hypercapnia, Phelan, *et al.* 2023 reported a CO_2_-dependent change in the cellular abundance of several amino acids—serine, methionine, threonine, valine, and proline. In THP-1 monocytes, a 4-h exposure to hypercapnia significantly reduced proline abundance, both in the absence and presence of LPS. Notably, RNA-sequencing analysis also revealed a CO_2_-dependent upregulation of pyrroline-5-carboxylate reductase 1 (*PYCR1*) in both absence and presence of LPS (92). PYCR1, a mitochondrial enzyme, is involved in the final step of proline synthesis catalyzing the conversion of pyrroline-5 carboxylate to proline (93). Taken together these findings offer further evidence of the hypercapnia-dependent impact on transcriptional and metabolite changes in pathways related to amino acid metabolism.

Proline is a key structural amino acid involved in collagen deposition, antioxidant activity, and immune responses (94). It is also associated with the development of monocyte-to-macrophage differentiation, which may influence an individual’s susceptibility to allergic asthma (95). Impaired PYCR1 function and reduced proline synthesis in hypercapnic immune cells may hinder wound healing and impair monocyte-to-macrophage differentiation in hypercapnia patients supporting the theory of hypercapnia-associated impairment of the innate immune system.

## Hypoxia and metabolism

In the presence of oxygen, ATP synthesis is mainly driven by the breakdown of glucose, glutamine, or fatty acids, which enter the TCA cycle and undergo OXPHOS within the mitochondria. In hypoxic conditions, cells can experience a metabolic crisis and take steps to transition away from a reliance on aerobic metabolism and toward anaerobic metabolism.

### Hypoxia and glycolysis

Upregulation of the glycolytic pathway in hypoxia is a classic example of metabolic plasticity in response to an energetic challenge. The orchestration of this metabolic change is predominantly regulated by HIFs, particularly HIF-1α (96). Hypoxia-induced upregulation of glycolysis is initially achieved allosterically and succeeded by HIF-1α-dependent transcriptional upregulation of glycolytic enzymes. In normoxic states, accumulation of ATP and/or citrate limits the activity of PFK to contribute to glycolytic ATP production. In hypoxic conditions, a reduction in mitochondrial ATP synthesis diminishes the allosteric suppression of the glycolytic pathway and promotes PFK phosphorylation of fructose-6-phosphate to fructose-1,6-bisphosphate, thus facilitating increased glycolytic flux (97). Moreover, pyruvate kinase is also regulated by a similar mechanism (98). Rapid, hypoxia-dependent allosteric regulation of glycolytic metabolism is subsequently followed by HIF-mediated transcriptional upregulation of glucose transporters and glycolytic enzymes.

The orchestral role of HIFs in the cellular response to hypoxia has been extensively characterized. HIF-1α specifically regulates system-level control of glucose metabolism and glycolysis (99). HIF-1α promotes glucose uptake by upregulating the expression membrane transporters such as glucose transporter 1 (GLUT1) (100). Hypoxia further enhances glycolytic flux through HIF-1α dependent transcriptional upregulation of all 10 glycolytic enzymes (28). Additionally, HIF-1α promotes the expression of both PDK1 (67), and LDH (68). Exposure of mouse embryonic fibroblasts to hypoxia for 48 and 72 h leads to a significant upregulation of PDK1 protein expression (67). This upregulation suppresses the conversion of pyruvate to acetyl-CoA by phosphorylating the pyruvate dehydrogenase complex, thereby redirecting pyruvate to be reduced by LDH and NADH, producing lactate and regenerating NAD⁺ (67). While the TCA produces a large abundance of NAD^+^, in states of hypoxia and downregulated aerobic metabolism, the necessary supply of NAD^+^ for glycolysis is achieved through the reduction of pyruvate to lactate (101).

Intriguingly, a recent study revealed that hypoxia evokes a nontranscriptional, spatial colocalization of glycolytic enzymes in intestinal epithelial cells, particularly following 24 h and 48 h of hypoxic exposure (102). The authors observed that both epithelial cells and human platelets, where transcription/translation was inhibited, exhibited the ability to induce glycolysis under hypoxia, indicating that a nontranscriptional element is also involved in the hypoxic-induced metabolic switch. The study demonstrated that hypoxia induces the formation of glycolytic enzyme complexes, which occurred independent of HIF-1α dependent transcription. The authors concluded that this may be a mechanism to enhance the efficiency of protein-protein and protein-metabolite interactions.

The application of mass spectrometry with ^13^C-isotope tracing further supports these observations. By tracing the catabolism of ^13^C-glucose through the metabolic pathways, glucose incorporation into the metabolites can be investigated, providing detailed insights into metabolic flux, revealing the activity and connectivity of the metabolic pathways. ^13^C-glucose tracing investigations conducted in bone marrow-derived endothelial progenitor cells for periods of up to 8 days revealed that hypoxia enhances glucose incorporation into glycolytic intermediates while suppressing glucose incorporation into the TCA cycle (103).

In summary, the hypoxic-induced switch from OXPHOS to O_2_-independent glycolysis is a redemptive adaptation to maintain ATP synthesis while mitigating the detrimental consequences of mitochondrial metabolism in the absence of oxygen (described later in this review). This metabolic switch serves as a compensatory strategy to try and maintain cell function and survival under hypoxic conditions. Understanding the regulation of the glycolytic pathway in this context is of extreme importance to understand health and disease in hypoxia.

### Hypoxia, mitochondrial function, the TCA cycle, and the electron transport chain

**Figure 3:**
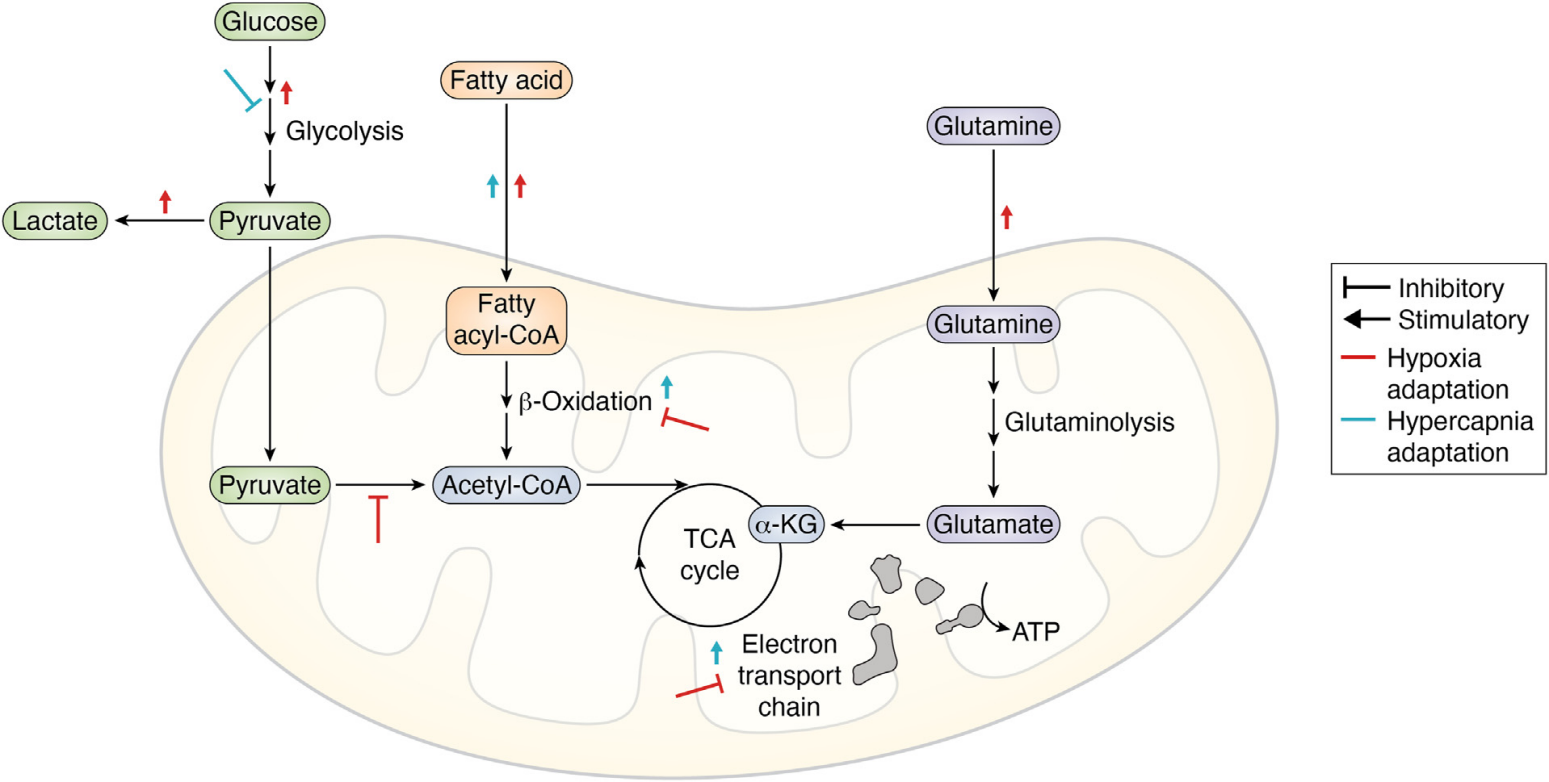
**Effect of hypoxia and hypercapnia on mitochondrial incorporation of glucose, fatty acids, and glutamine for metabolic processes.** This diagram illustrates the incorporation of glucose, fatty acids, and glutamine into mitochondrial metabolic processes. Glucose is depicted entering the cell, undergoing glycolysis to form pyruvate, which is then transported into the mitochondria for entry into the TCA cycle. Fatty acids are shown being transported into the mitochondria *via* the carnitine shuttle, undergoing β-oxidation to produce acetyl-CoA, which also contributes to the TCA cycle. Glutamine is converted to glutamate and subsequently to α-ketoglutarate, another intermediate of the TCA cycle. The figure also shows the effects of hypoxia and hypercapnia on these processes. *Red* stimulators and inhibitors represent the impact of hypoxia. *Blue* stimulators and inhibitors illustrate the effects of hypercapnia processes. Incorporation refers to the uptake and integration of specific molecules or nutrients into cellular or metabolic structures. α-KG, alpha-ketoglutarate; TCA cycle, tricarboxylic acid cycle.

In hypoxia, the HIF-1α dependent reduction in acetyl-CoA production from pyruvate significantly impairs TCA cycle flux. In the TCA cycle, citrate synthase catalyzes the formation of citrate from acetyl-CoA and oxaloacetate, marking the first reaction of the TCA cycle. Disruption of acetyl-CoA production and its reduced availability negatively impacts the entirety of subsequent reactions within the cycle, reducing production of ATP, GTP, NADH, and FADH_2_ in the mitochondria. Additionally, in states of hypoxia, O_2_ deprivation further impacts O_2_-dependent decarboxylation reactions of isocitrate and α-KG within the TCA cycle. This disruption impairs the production of mitochondrial NADH and TCA cycle intermediates, leading to a significant disturbance in the cycle's metabolic efficiency (Fig. 3).

Hypoxia influences cellular energetics by directly targeting mitochondrial function and OXPHOS capacity. Under normoxic conditions, mitochondria produce reducing equivalents or import them from the cytosol. For example, the NAD^+^/NADH shuttle allows for the exchange of H^+^ ions between cytosolic NADH and mitochondria NAD^+^. The hypoxia dependent downregulation of mitochondrial metabolism impacts downstream processes in the TCA cycle and ETC, as previously described. Hypoxia suppresses reducing equivalent formation, interferes with the stability of the ETC leading to the accumulation of ROS, which can damage mitochondria and DNA (104). Furthermore, hypoxia impacts the functionality of the ETC by modulating complex-activity in a HIF-1α dependent manner. The HIF-1α-dependent regulation of the ETC not only alters respiratory capacity of the ETC but also limits further ROS production, upholding cell survival in hypoxia. For example, the stabilization of HIF-1α suppresses complex I activity by transcriptionally upregulating the protein expression of NDUFA4L2, which in turn inhibits complex I activity after 18 h of hypoxia exposure. The decrease in complex I activity is a vital adaptation to hypoxia, as it suppresses mitochondrial superoxide production, a type of ROS (105).

An additional hypoxia-dependent decrease in ETC activity is also achieved by specifically targeting complex IV, which is critically reliant on adequate oxygen availability to convert H^+^ ions and molecular oxygen using the electron supply of the ETC to form H_2_O. The absence of oxygen availability has been shown to significantly diminish maximal functionality of complex IV (106). Beyond oxygen deprivation, hypoxia affects complex IV activity *via* transcriptional regulation. HIF-1α mediates the transcriptional upregulation of cytochrome c oxidase IV isoform 2 (COX4-2) and the mitochondrial Lon protease. COX4-2 replaces COX4-1 as COX4-1 is targeted by mitochondrial Lon for proteasomal degradation. HIF-1α dependent upregulation of COX4-2 enhances the complex IV’s electron transfer efficiency in attempt to sustain H_2_O formation and ATP production (107).

Mitochondria respond to hypoxia in an attempt to minimize ROS accumulation and effectively regulate mitochondrial quality and function. In hypoxia, these processes, including changes in mitochondrial dynamics and mitophagy, are activated as part of the mitochondrial response (108). For instance, hypoxia-induced mitochondrial dysfunction leads to the accumulation of short-form optic atrophy factor 1 (sOPA1) in AC16 human cardiomyocytes (109). Accumulation of short-form optic atrophy factor 1 inhibits mitochondrial fusion and promotes mitochondrial fission, resulting in the formation of smaller, round mitochondria. Increased rates of mitochondrial fission lead to the formation of smaller, more discrete mitochondria promoting mitophagy (110). Hypoxic-induced mitochondrial fission serves as a protective mechanism by promoting the removal of damaged mitochondria through mitophagy, helping to maintain a healthy mitochondrial network (108). Enhanced mitochondrial fission is likely a protective response to hypoxia-induced mitochondrial damage. Various other fission-associated proteins, mechanisms, and factors also regulate mitochondrial dynamics under hypoxia, forming a complex regulatory network which are extensively researched by the following (109, 111).

### Hypoxia and lipid metabolism

Fatty acids can be acquired through external uptake or synthesized endogenously *via* the DNL pathway. Traditionally, fatty acids serve as a nutrient source that supports energy production, membrane formation, and the synthesis of signaling molecules but it is well-established that fatty acids are essential for additional cellular functions. The oxidation of fatty acids occurs in mitochondria and requires oxygen. Under hypoxic conditions, the decrease in mitochondrial activity significantly impacts the metabolism and fate of free fatty acids. Fatty acid metabolism, like other pathways, adapts to the oxygen-deficient environment by prioritizing the use of fatty acids for processes other than aerobic metabolism.

The production of fatty acid precursors is supported through HIF-1α-dependent stimulation of reductive glutamine metabolism, as discussed later in this review. Hypoxia-induced upregulation of glutaminolysis plays a crucial role in providing fuel for DNL synthesis by facilitating the incorporation of glutamine into the TCA cycle. Additionally, HIF-1α increases the abundance of α-KG by upregulating the HIF-1α target glutaminase-1 (GLS1), accelerating the synthesis of glutamate-derived α-KG. After 72 h of hypoxic exposure in A549 cells, glutamine-derived α-KG undergoes reductive carboxylation *via* IDH1 to form citrate, rather than being decarboxylated to succinyl-CoA. This citrate is then utilized for lipid synthesis through the DNL pathway (112).

Impairments in fatty acid metabolism and the accumulation of free fatty acids can result in lipotoxicity. Typically, the esterification of fatty acids into triacyl glycerides (TAGs) and their storage in lipid droplets (LDs) help protect cancer cells from lipotoxicity (Bensaad *et al.* 2014) while serving as a significant energy reserve (113). The formation of LDs not only shields hypoxic cells from lipotoxicity but also mitigates the adverse effects of free radical production on cells during cycles of hypoxia (114, 115).

Cardiomyocytes exposed to hypoxia exhibit a HIF-1α-dependent increase in extracellular fatty acid uptake and TAG synthesis. This upregulation of fatty acid uptake and TAG synthesis is driven by HIF-1α-dependent activation of peroxisome proliferator-activated receptor gamma (116). Additionally, the stabilization of HIF-1α following 48 h of hypoxic exposure enhances extracellular fatty acid uptake through increased mRNA expression of fatty acid binding proteins (FABPs) 3 and 7 in U87 and MCF-7 cells (Bensaad et al 2014). HIF-1α stabilization also upregulates the protein expression of low-density lipoprotein receptor-related protein 1 in vascular smooth muscle cells and the very low-density lipoprotein receptor in cardiomyocytes (117, 118). The increased expression of these receptors promotes endocytosis of lipoproteins. The link between dysregulated low-density lipoprotein (LDL) content, dyslipidaemia, and cardiovascular related diseases such as atherosclerosis is well-established in current extensive clinical and epidemiological studies and reviews such as those by (119, 120).

Bensaad *et al.* 2014 observed that FABPs promote fatty acid uptake following 48 h exposure of hypoxia. This ultimately led to the accumulation of LDs as an adaptive response to support cell survival. In breast cancer MCF10a cells, higher LD levels were associated with improved survival under hypoxic conditions. *siRNA* knock down of adipose differentiation-related protein *(ADRP), FABP3,* and *FABP7* revealed a significant decrease of spheroid size in 3D models which mimic hypoxic tumor microenvironments. The authors concluded that LD formation in response to hypoxia is a critical adaptation that supports cell growth. Lastly, they proposed that this process serves as a protective mechanism against ROS accumulation by diverting free fatty acids into the TAG synthesis pathway, thereby reducing their entry into ROS-generating pathways.

Moreover, acylglycerol-3-phosphate acyltransferase 2 (AGPAT2), also known as lysophosphatidic acid acyltransferase β (LPAATβ), is an intermediate enzyme within the pathway for the TAG biosynthesis. AGPAT2 catalyzes the conversion of lysophosphatidic acid to phosphatidic acid—two intermediates of the TAG biosynthesis pathway. This enzyme has been shown to be a direct HIF-1α target (121). Furthermore, total AGPAT2 protein expression was upregulated following 8 and 24 h of hypoxia exposure in Huh7 and HeLa cells. Additionally, *siRNA-*mediated knockdown of HIF-1α impaired AGPAT2 expression, suggesting that HIF-1α regulates hypoxia-induced LD accumulation by targeting the TAG biosynthesis pathway (121). The authors concluded that their findings demonstrate that AGPAT2 plays a significant role in orchestrating the hypoxic-dependent change in lipid metabolism to facilitate cell adaptation and survival under low oxygen conditions. For further insights into the effects of hypoxia and HIF-1α-mediated responses in lipid metabolism, refer to the extensive review by Mylonis *et al.* (122).

Hypoxic microenvironments are associated with the proliferation of arterial smooth muscle cells (SMCs) as people age (117). Conditions such as hypertension, hypercholesterolemia, and atherosclerosis lead to arterial wall thickening due to atherosclerotic plaque buildup. Hypoxic environments are characteristic of advanced atherosclerotic lesions and are linked to angiogenesis and thrombus formation (123). In localized hypoxia within SMCs, there is an upregulation of fatty acid uptake, lipid vesicle formation, angiogenesis, inflammation, and lipid deposition (124). Additionally, HIF-1α accumulation in macrophages promotes foam cell formation and atherosclerosis, as foam cells form when a macrophage’s intracellular lipid content surpasses their ability to maintain lipid homeostasis, impacting immune function (125).

Research indicates that in the early stages of hypoxia (6 and 24 h), HIF-1α expression upregulates low density lipoprotein receptor-related protein 1 (LRP1) expression in vascular SMCs, a relationship seen in advanced human atherosclerotic lesions, suggesting that hypoxic vascular SMCs contribute to high HIF-1α levels, which are associated with LDL endocytosis (117). Another study found that 8 h of hypoxia exposure induces lipid accumulation in HL-1 cardiomyocytes by upregulating very low-density lipoprotein receptor protein expression *via* HIF-1α, thereby promoting very low-density lipoprotein endocytosis (118).

Hypoxia stabilization of HIF-1α upregulates fatty acid transporters and lipid biosynthesis enzymes to facilitate fatty acid uptake, DNL, TAG synthesis, and LD formation. In hypoxic conditions, lipid vesicle utilization for energy supply is likely reduced because β-oxidation—the process of breaking down fatty acids for energy—is suppressed due to oxygen deprivation. As a result, cells shift away from fatty acid oxidation and rely on alternative energy pathways, such as glycolysis, reducing the reliance on lipid vesicles as an energy source.

**Figure 4:**
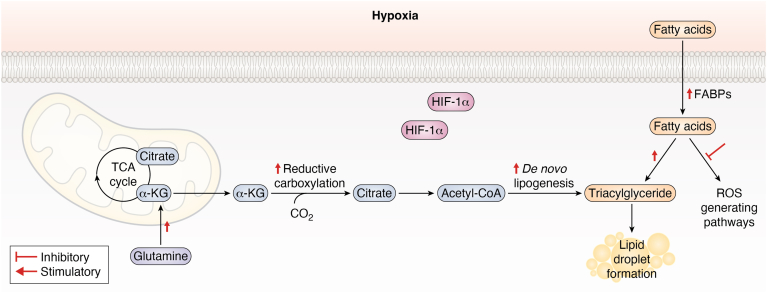
**Hypoxia-induced *de novo* lipogenesis and lipid droplet formation.** This diagram illustrates the effect of hypoxia on mitochondrial metabolic processes, specifically highlighting its role in driving *de novo* lipogenesis and lipid droplet formation. Under hypoxic conditions, cells redirect metabolic flux toward lipogenesis, promoting the synthesis of fatty acids and their storage in lipid droplets. *Red* stimulators and inhibitors represent the impact of hypoxia. α-KG, alpha-ketoglutarate; CO_2_, carbon dioxide; FABP, fatty acid binding protein; HIF-1α, hypoxia-inducible factor 1α; TCA cycle, tricarboxylic acid cycle.

This metabolic adaptation helps cells survive under low oxygen conditions by preventing lipotoxicity, as excess fatty acids are stored in LDs rather than being oxidized. However, this also leads to excessive lipid accumulation within cells, contributing to foam cell formation—a key event in the initial stages of atherosclerosis. Over time, this process accelerates the progression of atherosclerotic plaques, increasing the risk of cardiovascular diseases. Thus, while hypoxia-driven changes in lipid metabolism help cells adapt and survive, they also play a significant role in the development and worsening of cardiovascular conditions (Fig. 4).

### Hypoxia and amino acid metabolism

Glutamine, a nonessential amino acid, plays a versatile role in cellular metabolism. Following glutaminolysis, glutamine can be incorporated into the TCA cycle or play a role in amino acid synthesis. Glutamine is considered as a critical fuel source in cancer cell metabolism, with glutamine deprivation shown to suppress tumor growth (65, 126, 127). In hypoxic conditions, glutaminolysis is enhanced by the upregulation of glutamine transporters and mitochondrial enzymes that convert glutamine into glutamate, highlighting its role as a primary fuel source, especially during processes such as cancer cell proliferation (59, 128). This upregulation of glutaminolysis is crucial for supporting mitochondrial function and TCA cycle intermediate production, when glucose availability is diminished (128). HIF-2α increases the expression of the SLC1A5 glutamine transporter, promoting glutamine uptake into the mitochondria. Furthermore, GLS1 protein expression is upregulated in response to hypoxia-induced HIF-1α following 48 h of exposure, promoting the incorporation of glutamine into the TCA cycle (129). Moreover, the application of mass spectrometry employing ^13^C isotope tracing verified that glucose incorporation into TCA cycle metabolites—citrate, α-KG, fumarate, and malate—is supressed in hypoxia. This specific study also revealed a hypoxic-dependent decrease in expression of three key TCA cycle enzymes: citrate synthase, IDH3, and α-KG dehydrogenase. The application of mass spectrometry employing ^13^C-glutamine, revealed that hypoxic-induced upregulation of glutamine incorporation into the TCA cycle occurred following 24 and 48 h of hypoxic exposure in several cell lines (130, 131, 132).

As previously mentioned, hypoxia-driven upregulation of glutaminolysis fuels DNL, a process that converts simple molecules into complex lipids to support cellular function and proliferation (35). After 72 h of hypoxic exposure in A549 cells, glutamine-derived α-KG undergoes reductive carboxylation *via* IDH1 to form citrate (112), which then supports DNL, promoting tumor progression (128).

Additionally, glutamine incorporation into the mitochondria is necessary for glutathione synthesis which is a vital component of cellular protection against oxidative stress (133). It has been reported that HIF-1α promotes the synthesis of GSH through the upregulation of GLS1 protein expression, thereby mitigating ROS damage during hypoxia (134, 135). In triple-negative breast cancer cells, HIF-1α stabilization was also found to promote GSH synthesis by upregulating the expression of the glutamate-cysteine ligase modifier subunit, a key regulator in glutathione biosynthesis. Furthermore, knockdown of *HIF-1α via* shRNA reduced glutamate-cysteine ligase modifier mRNA and protein expression after 72 h of hypoxia exposure, suggesting that this HIF-1α-dependent pathway serves as a protective mechanism against hypoxia-induced ROS production from the electron transport chain (136).

Glutamic-oxaloacetic transaminase 1 (GOT1, cytosolic) and 2 (GOT2, mitochondrial) or alternatively known as aspartate aminotransferase 1 and 2, facilitate the interconversion of oxaloacetate and glutamate into aspartate and α-KG. Meléndez-Rodríguez, *et al.* used various cell models to study HIF-1α transcriptional activity under normoxia, particularly in VHL-deficient 786-O cells, which have constitutive HIF-1α stabilization (137). They compared control 786-O cells with those overexpressing HIF-1α and a mutant version with a four-amino-acid substitution in the bHLH domain that impairs HIF-1α transcriptional ability. Additionally, they used UCDMel-ΔH melanoma cells, comparing controls to those with proline residue mutations that inhibit VHL-mediated degradation and a bHLH mutant variant, similar to the one used in 786-O cells. HIF-1α overexpression in 786-O and melanoma cells significantly downregulated the expression of GOT1 and GOT2, resulting in the suppression of aspartate biosynthesis. The HIF-1α-dependent reduction in aspartate biosynthesis, caused by the suppression of GOT1 and GOT2, is a key metabolic adaptation that helps limit cell proliferation. The authors examined how hypoxia-induced stabilization of HIF-1α affects the expression of GOT1 and GOT2 in G55 human glioma cells, which possess the VHL protein and lack constitutive HIF-1α activation. They discovered that stabilization of HIF-1α under hypoxic conditions also led to a reduction in GOT1 and GOT2 protein levels. Silencing *HIF-1α* with *siRNA* in the G55 glioma cell line reversed this hypoxia-induced suppression of GOT1 and GOT2 expression. This finding suggests that HIF-1α is responsible for the decrease in aspartate biosynthesis, in addition to its role in inhibiting glutamine oxidation under oxygen deprivation. Additionally, the authors revealed that ^13^C -glutamine incorporation into both fumarate and malate were significantly decreased in a HIF-1α dependent manner in both cell lines (137). Taken together these data suggest a tumor suppressor role for HIF-1α, whereby stabilization of HIF-1α actively limits aspartate biosynthesis by suppressing cytosolic GOT1 and mitochondrial GOT2, and further reduces glutamine incorporation into TCA cycle intermediates.

Mitochondria are crucial for regulating the cellular NADH/NAD^+^ ratio. Hypoxia disrupts this ratio, leading to TCA cycle dysregulation and triggering alternative redox-active pathways to regenerate NAD^+^ (138). NAD^+^ acts as an electron carrier in redox reactions, essential for maintaining redox balance and energy metabolism. Proline synthesis helps maintain cellular redox states by oxidizing NADH to keep NAD^+^ levels stable. Loss of PYCR1 activity affects the NADH/NAD^+^ ratio, underscoring its role in mitochondrial NAD^+^ provision (139). Westbrook *et al.* demonstrated that 24 h exposure of hypoxia significantly increases intracellular proline abundance in several cell lines (triple-negative breast cancer (SUM159PT and HCC1806), medulloblastoma (ONS), and bone marrow stromal cells (HS-5)). Furthermore, the application of ^13^C-isotope tracing in SUM159PT cells revealed that hypoxia significantly increases ^13^C-glutamine incorporation into proline. A role for PYCR1 in this process was inferred by experiments performed in SUM159PT cells where PYCR1 was knocked down using siRNA. These siPYCR1 cells exhibited a marked reduction in intracellular proline derived from glutamine, further emphasizing the crucial role of PYCR1 in hypoxic conditions. The authors concluded that PYCR1 is essential for maintaining the mitochondrial NADH/NAD^+^ ratio under hypoxia, as the oxidation of NADH to NAD^+^ is vital for cellular redox balance.

**Figure 5:**
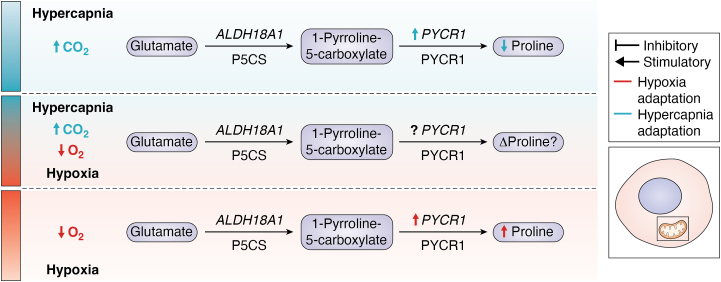
**The effect of hypercapnia and hypoxia on proline synthesis.** This figure features three schematics illustrating the contrasting effects of hypercapnia and hypoxia on cellular proline levels. The top schematic highlights how hypercapnia suppresses proline levels and affects PYCR1 mRNA. The bottom schematic depicts the impact of hypoxia, which promotes an increase in proline. The central schematic explores the interplay of a hypercapnic/hypoxic microenvironment, commonly observed in tumors, with a focus on how these opposing conditions could potentially influence proline metabolism. *Red* stimulators and inhibitors represent the impact of hypoxia. *Blue* stimulators and inhibitors illustrate the effects of hypercapnia. APYCR1, pyrroline-5-carboxylate reductase 1; CO₂, carbon dioxide; LDH18A1, aldehyde dehydrogenase 18 family member A1; O₂, oxygen; P5CS, pyrroline-5-carboxylate synthase.

Under hypoxic conditions, HIF-1α plays a pivotal role in modulating amino acid metabolism to sustain cellular function and survival while also altering the fate of glutamine metabolism. HIF-1α suppresses key enzymes in the TCA cycle, leading to reduced aspartate synthesis, while promoting proline synthesis in a PYCR1 dependent manner (Fig. 5).

The stabilization of HIF-1α further promotes glutathione synthesis, a vital antioxidant defense against oxidative stress, which is increased in hypoxia. This coordinated response with respect to amino acid metabolism underscores the adaptability of cellular metabolism in hypoxia and highlights the potential for targeting these pathways in therapeutic strategies.

Lastly, 48 h exposure of hypoxia has been shown to increase both *mRNA* and protein levels of L-type amino acid transporter 1, a branched-chain amino acid (BCAA) transporter, and branched-chain aminotransferase 1 (BCAT1), which catalyzes the first step in BCAA catabolism, in glioblastoma cells. HIF-1α and HIF-2α are involved in the upregulation of L-type amino acid transporter 1, while only HIF-1α drives BCAT1 expression. CRISPR/Cas9 knockout of HIF-1α or HIF-2α reduced hypoxia-induced glutamate production from BCAAs. The authors also found that inhibition of BCAT1 in U-251MG cells diminished glioblastoma cell growth following 7 days exposure of hypoxia, and concluded that hypoxic-induced reprogramming of BCAA metabolism is a key adaptation to maintain glutamate abundance to support cell and tumor growth under hypoxic conditions (140).

## Conclusion

In conclusion, the equilibrium between O_2_ and CO_2_ is vital for maintaining cellular and systemic homeostasis. These gases play essential roles in respiration and metabolism, and their balance is crucial for efficient energy production and cellular function. Dynamic regulation of O_2_ and CO_2_ directly impacts cellular metabolism and mitochondrial activity, particularly under conditions like hypoxia and hypercapnia. While the impact of hypoxia is well-documented, the physiological consequences associated with hypercapnia are less thoroughly explored.

**Figure 6:**
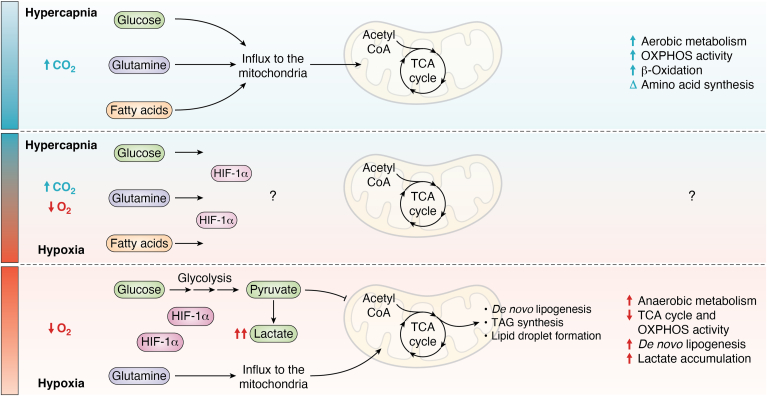
**Comparative schematics of the effects of hypercapnia and hypoxia on cellular metabolism.** This figure features three schematics illustrating the effects of hypercapnia and hypoxia on cellular metabolism. The top schematic depicts the influence of hypercapnia on metabolic pathways, while the bottom schematic details how hypoxia affects metabolic pathways. The central schematic questions the potential metabolic consequences of a hypercapnic/hypoxic microenvironment, commonly observed in tumors. The central diagram shows a reduction in the size of HIF-1α, indicating the suppression of HIF-1α stabilization under hypercapnic conditions, as reported by (12). CO₂, carbon dioxide; HIF-1α, hypoxia-inducible factor 1α; O₂, oxygen; OXPHOS, oxidative phosphorylation; TAG, triacyl glyceride; TCA cycle, tricarboxylic acid cycle.

Since hypoxia and hypercapnia frequently coexist in both physiological and cancerous microenvironments, an important question arises: is there a crosstalk between CO_2_ and O_2_, and if so, how does it influence cellular function? This potential CO_2_-O_2_ interaction could have profound implications for cellular metabolism, mitochondrial dynamics, and immune responses, especially given the contrasting effects these gases exert on metabolic pathways. Understanding this interplay is essential for deciphering how cells adapt to combined hypoxic and hypercapnic conditions, with implications for both health and disease. This is especially relevant as each condition alone has contrasting effects on metabolic pathways. Peripheral blood mononuclear cells from COPD patients showed significantly reduced HIF-1α protein expression in response to 24 h of hypoxia when compared to healthy individuals (141), possibly indicating a counter-regulatory role for hypercapnia. Selfridge, *et al.* demonstrated that hypercapnia suppresses both HIF-1α and HIF-2α protein expression, and studies focused on hypercapnia have revealed unique metabolic adaptations that differ from those driven by hypoxia and HIF-1α (Figs. 1, 3, 5, and 6). Thus, an emerging concept is that hypercapnia could counter-regulate HIF-1α and HIF-2α activity. The impact of hypercapnia in different systems is likely dictated by the local environment. In the tumor microenvironment, where hypoxia and hypercapnia coexist, hypercapnia could potentially attenuate hypoxia-induced HIF-α expression, counter-regulating HIF-1α and HIF-2α activity, and influence tumor cell metabolism/chemoresistance. This may be different to what occurs in situations where hypercapnia is a consequence of type 2 respiratory failure caused by respiratory pump failure, or increased CO_2_ production (142) which occurs without the concurrent hypoxic conditions.

Future research should focus on elucidating the mechanisms of CO_2_-O_2_ interplay in health and disease. Interestingly, a hypercapnia inducible factor analogous to HIF has been hypothesized. The authors propose that such a factor could be responsible for the regulation of hundreds of genes in response to elevated CO_2_, in a similar manner to how HIF regulates hundreds of genes in response to hypoxia. They further speculate that there may be a “certain number of specific and identical (overlapping) genes and metabolic pathways controlled by hypercapnia inducible factor and HIF” (143). This will be relevant in the situations of hypercapnia and hypoxia outlined above but also in other hypoxic-hypercapnic environments such as the burrows of naked mole rats. These mammals can tolerate extreme gaseous environments of O_2_ (surviving anoxia much longer than mice) (144) and CO_2_ (with animals having seizures when CO_2_ levels are too low) (145), have altered metabolism, markedly increased longevity compared to mice and resistance to aging associated pathologies including cancer (146). Thus, the question as to how does a hypercapnic/hypoxic microenvironment influence cellular metabolic profiles and immune function is a fundamental question for all animals given the gaseous makeup of the Earth’s atmosphere. Gaining a deeper insight into this area is essential for developing our understanding of the role of CO_2_ and O_2_; separately and together; in health and disease (Fig. 6).

## Conflict of interest

The authors declare that they have no conflicts of interest with the contents of this article.

## References

[1] Pahal P., Hashmi M.F., Sharma S. (2018).

[2] Feher J.J. (2017).

[3] Hill R.J., Davis R.W. (1967). The pK of specific groups of proteins: I. The α-AMINO group of the α chain of human CO-hemoglobin. J. Biol. Chem..

[4] Perutz M.F. (1983). Species adaptation in a protein molecule. Mol. Biol. Evol..

[5] Viniol C., Vogelmeier C.F. (2018). Exacerbations of COPD. Eur. Respir. Rev..

[6] Phelan D.E., Mota C., Strowitzki M.J., Shigemura M., Sznajder J.I., Crowe L. (2023). Hypercapnia alters mitochondrial gene expression and acylcarnitine production in monocytes. Immunol. Cell Biol..

[7] Rawat D., Modi P., Sharma S. (2022).

[8] Kikuchi R., Iwai Y., Tsuji T., Watanabe Y., Koyama N., Yamaguchi K. (2019). Hypercapnic tumor microenvironment confers chemoresistance to lung cancer cells by reprogramming mitochondrial metabolism in vitro. Free Radic. Biol. Med..

[9] Helmlinger G., Sckell A., Dellian M., Forbes N.S., Jain R.K. (2002). Acid production in glycolysis-impaired tumors provides new insights into tumor metabolism. Clin. Cancer Res..

[10] Benner A., Lewallen N.F., Sharma S. (2023). StatPearls.

[11] Chen Z., Han F., Du Y., Shi H., Zhou W. (2023). Hypoxic microenvironment in cancer: molecular mechanisms and therapeutic interventions. Signal. Transduct. Targeted Therapy.

[12] Selfridge A.C., Cavadas M.A., Scholz C.C., Campbell E.L., Welch L.C., Lecuona E. (2016). Hypercapnia suppresses the HIF-dependent adaptive response to hypoxia. J. Biol. Chem..

[13] Cummins E.P., Strowitzki M.J., Taylor C.T. (2020). Mechanisms and consequences of oxygen and carbon dioxide sensing in mammals. Physiol. Rev..

[14] Laserna E., Sibila O., Aguilar P.R., Mortensen E.M., Anzueto A., Blanquer J.M. (2012). Hypocapnia and hypercapnia are predictors for ICU admission and mortality in hospitalized patients with community-acquired pneumonia. Chest.

[15] Jaitovich A., Angulo M., Lecuona E., Dada L.A., Welch L.C., Cheng Y. (2015). High CO2 levels cause skeletal muscle atrophy via AMP-activated kinase (AMPK), FoxO3a protein, and muscle-specific Ring finger protein 1 (MuRF1). J. Biol. Chem..

[16] Gao S., Zhang Z., Brunelli A., Chen C., Chen C., Chen G. (2017). The Society for Translational Medicine: clinical practice guidelines for mechanical ventilation management for patients undergoing lobectomy. J. Thorac. Dis..

[17] Laffey J.G., Kavanagh B.P. (1999). Carbon dioxide and the critically ill—too little of a good thing?. Lancet.

[18] Lorimer G.H., Miziorko H.M. (1980). Carbamate formation on the epsilon-amino group of a lysyl residue as the basis for the activation of ribulosebisphosphate carboxylase by CO2 and Mg2+. Biochemistry.

[19] King D.T., Zhu S., Hardie D.B., Serrano-Negrón J.E., Madden Z., Kolappan S. (2022). Chemoproteomic identification of CO2-dependent lysine carboxylation in proteins. Nat. Chem. Biol..

[20] Matthew J.B., Wittebort R.J., Hayes D.F., Rothgeb T.M., Gurd R.S., Gurd F.R.N. (1977). Reaction of carbon-dioxide with amino-groups in model systems, peptide hormones, and hemoglobin. Abstr. Pap. Am. Chem. Soc..

[21] Linthwaite V.L., Janus J.M., Brown A.P., Wong-Pascua D., O’Donoghue A.M.C., Porter A. (2018). The identification of carbon dioxide mediated protein post-translational modifications. Nat. Commun..

[22] Linthwaite V.L., Pawloski W., Pegg H.B., Townsend P.D., Thomas M.J., So V.K.H. (2021). Ubiquitin is a carbon dioxide-binding protein. Sci. Adv..

[23] Nijjar S., Brotherton D., Butler J., Dospinescu V.M., Gannon H.G., Linthwaite V. (February 5, 2025). Multiple carbamylation events are required for differential modulation of Cx26 hemichannels and gap junctions by CO_2_. J. Physiol..

[24] Lee J.W., Ko J., Ju C., Eltzschig H.K. (2019). Hypoxia signaling in human diseases and therapeutic targets. Exp. Mol. Med..

[25] Luo Z., Tian M., Yang G., Tan Q., Chen Y., Li G. (2022). Hypoxia signaling in human health and diseases: implications and prospects for therapeutics. Signal. Transduct. Targeted Ther..

[26] Eales K.L., Hollinshead K.E., Tennant D.A. (2016). Hypoxia and metabolic adaptation of cancer cells. Oncogenesis.

[27] Lee P., Chandel N.S., Simon M.C. (2020). Cellular adaptation to hypoxia through hypoxia inducible factors and beyond. Nat. Rev. Mol. Cell Biol..

[28] Taylor C.T., Scholz C.C. (2022). The effect of HIF on metabolism and immunity. Nat. Rev. Nephrol..

[29] Hu C.J., Wang L.Y., Chodosh L.A., Keith B., Simon M.C. (2003). Differential roles of hypoxia-inducible factor 1α (HIF-1α) and HIF-2α in hypoxic gene regulation. Mol. Cell Biol..

[30] Semenza G.L. (2010). HIF-1: upstream and downstream of cancer metabolism. Curr. Opin. Genet. Dev..

[31] Strowitzki M.J., Cummins E.P., Taylor C.T. (2019). Protein hydroxylation by hypoxia-inducible factor (HIF) hydroxylases: unique or ubiquitous?. Cells.

[32] Selak M.A., Armour S.M., MacKenzie E.D., Boulahbel H., Watson D.G., Mansfield K.D. (2005). Succinate links TCA cycle dysfunction to oncogenesis by inhibiting HIF-α prolyl hydroxylase. Cancer Cell.

[33] Dunn J., Grider M.H. (2023).

[34] Prieß M., Göddeke H., Groenhof G., Schäfer L.V. (2018). Molecular mechanism of ATP hydrolysis in an ABC transporter. ACS Cent. Sci..

[35] Wu S., Näär A.M. (2019). A lipid-free and insulin-supplemented medium supports De Novo fatty acid synthesis gene activation in melanoma cells. PLoS One.

[36] Akram M. (2013). Mini review on glycolysis and cancer. J. Cancer Edu..

[37] Lunt S.Y., Vander Heiden M.G. (2011). Aerobic glycolysis: meeting the metabolic requirements of cell proliferation. Annu. Rev. Cell Dev. Biol..

[38] Chaudhry R., Varacallo M. (2018).

[39] Picard M., Shirihai O.S. (2022). Mitochondrial signal transduction. Cell Metab..

[40] Herzig S., Shaw R.J. (2018). AMPK: guardian of metabolism and mitochondrial homeostasis. Nat. Rev. Mol. Cell Biol..

[41] Gwinn D.M., Shackelford D.B., Egan D.F., Mihaylova M.M., Mery A., Vasquez D.S. (2008). AMPK phosphorylation of raptor mediates a metabolic checkpoint. Mol. Cell.

[42] Cantó C., Auwerx J. (2009). PGC-1α, SIRT1 and AMPK, an energy sensing network that controls energy expenditure. Curr. Opin. Lipidol..

[43] Martínez-Reyes I., Chandel N.S. (2020). Mitochondrial TCA cycle metabolites control physiology and disease. Nat. Commun..

[44] Arnold P.K., Finley L.W. (2023). Regulation and function of the mammalian tricarboxylic acid cycle. J. Biol. Chem..

[45] Alabduladhem T.O., Bordoni B. (2022). StatPearls.

[46] Murphy M.P., Chouchani E.T. (2022). Why succinate? Physiological regulation by a mitochondrial coenzyme Q sentinel. Nat. Chem. Biol..

[47] Okuno D., Iino R., Noji H. (2011). Rotation and structure of F o F 1-ATP synthase. J. Biochem..

[48] Scialò F., Fernández-Ayala D.J., Sanz A. (2017). Role of mitochondrial reverse electron transport in ROS signalling potential roles in health and disease. Front. Physiol..

[49] Sun C., Benlekbir S., Venkatakrishnan P., Wang Y., Hong S., Hosler J. (2018). Structure of the alternative complex III in a supercomplex with cytochrome oxidase. Nature.

[50] Yoshida M., Muneyuki E., Hisabori T. (2001). ATP synthase—a marvellous rotary engine of the cell. Nat. Rev. Mol. Cell Biol..

[51] Zhao R.Z., Jiang S., Zhang L., Yu Z.B. (2019). Mitochondrial electron transport chain, ROS generation and uncoupling. Int. J. Mol. Med..

[52] Nolfi-Donegan D., Braganza A., Shiva S. (2020). Mitochondrial electron transport chain: oxidative phosphorylation, oxidant production, and methods of measurement. Redox Biol..

[53] Chandel N.S. (2021). Amino acid metabolism. Cold Spring Harb. Perspect. Biol..

[54] Knottnerus S.J., Bleeker J.C., Wüst R.C., Ferdinandusse S., IJlst L., Wijburg F.A. (2018). Disorders of mitochondrial long-chain fatty acid oxidation and the carnitine shuttle. Rev. Endocr. Metab. Disord..

[55] Talley J.T., Mohiuddin S.S. (2020).

[56] Curthoys N.P., Watford M. (1995). Regulation of glutaminase activity and glutamine metabolism. Annu. Rev. Nutr..

[57] Stine Z.E., Dang C.V. (2020). Glutamine skipping the Q into mitochondria. Trends Molecular Medicine.

[58] Wang J.B., Erickson J.W., Fuji R., Ramachandran S., Gao P., Dinavahi R. (2010). Targeting mitochondrial glutaminase activity inhibits oncogenic transformation. Cancer Cell.

[59] Yoo H.C., Park S.J., Nam M., Kang J., Kim K., Yeo J.H. (2020). A variant of SLC1A5 is a mitochondrial glutamine transporter for metabolic reprogramming in cancer cells. Cell Metab..

[60] Mullen A.R., Hu Z., Shi X., Jiang L., Boroughs L.K., Kovacs Z. (2014). Oxidation of alpha-ketoglutarate is required for reductive carboxylation in cancer cells with mitochondrial defects. Cell Rep..

[61] Yang L., Venneti S., Nagrath D. (2017). Glutaminolysis: a hallmark of cancer metabolism. Annu. Rev. Biomed. Eng..

[62] Chandel N.S. (2021). Lipid metabolism. Cold Spring Harb. Perspect. Biol..

[63] Pirovich D.B., Da’dara A.A., Skelly P.J. (2021). Multifunctional fructose 1, 6-bisphosphate aldolase as a therapeutic target. Front. Mol. Biosci..

[64] Li M., Zhang C.S., Zong Y., Feng J.W., Ma T., Hu M. (2019). Transient receptor potential V channels are essential for glucose sensing by aldolase and AMPK. Cell Metab..

[65] Zhang C.S., Hawley S.A., Zong Y., Li M., Wang Z., Gray A. (2017). Fructose-1, 6-bisphosphate, and aldolase mediate glucose sensing by AMPK. Nature.

[66] Semenza G.L., Roth P.H., Fang H.M., Wang G.L. (1994). Transcriptional regulation of genes encoding glycolytic enzymes by hypoxia-inducible factor 1. J. Biol. Chem..

[67] Kim J.W., Tchernyshyov I., Semenza G.L., Dang C.V. (2006). HIF-1-mediated expression of pyruvate dehydrogenase kinase: a metabolic switch required for cellular adaptation to hypoxia. Cell Metab..

[68] Semenza G.L., Jiang B.H., Leung S.W., Passantino R., Concordet J.P., Maire P. (1996). Hypoxia response elements in the aldolase A, enolase 1, and lactate dehydrogenase A gene promoters contain essential binding sites for hypoxia-inducible factor 1. J. Biol. Chem..

[69] Vohwinkel C.U., Lecuona E., Sun H., Sommer N., Vadász I., Chandel N.S. (2011). Elevated CO2 levels cause mitochondrial dysfunction and impair cell proliferation. J. Biol. Chem..

[70] Al-Khallaf H. (2017). Isocitrate dehydrogenases in physiology and cancer: biochemical and molecular insight. Cell Biosci..

[71] Goldberg J., Currais A., Prior M., Fischer W., Chiruta C., Ratliff E. (2018). The mitochondrial ATP synthase is a shared drug target for aging and dementia. Aging cell.

[72] Li S., Gao D., Jiang Y. (2019). Function, detection and alteration of acylcarnitine metabolism in hepatocellular carcinoma. Metabolites.

[73] Zhang J., Pavlova N.N., Thompson C.B. (2017). Cancer cell metabolism: the essential role of the nonessential amino acid, glutamine. EMBO J..

[74] Balnis J., Korponay T.C., Jaitovich A. (2020). AMP-activated protein kinase (AMPK) at the crossroads between CO2 retention and skeletal muscle dysfunction in chronic obstructive pulmonary disease (COPD). Int. J. Mol. Sci..

[75] Balnis J., Lee C.G., Elias J.A., Jaitovich A. (2020). Hypercapnia-driven skeletal muscle dysfunction in an animal model of pulmonary emphysema suggests a complex phenotype. Front. Physiol..

[76] Mukund K., Subramaniam S. (2020). Skeletal muscle: a review of molecular structure and function, in health and disease. Wiley Inter. Rev. Syst. Biol. Med..

[77] Zumbaugh M.D., Johnson S.E., Shi T.H., Gerrard D.E. (2022). Molecular and biochemical regulation of skeletal muscle metabolism. J. Anim. Sci..

[78] Hussain S.N.A., Sandri M. (2013). Role of autophagy in COPD skeletal muscle dysfunction. J. Appl. Physiol. (1985).

[79] Chapman N.M., Boothby M.R., Chi H. (2020). Metabolic coordination of T cell quiescence and activation. Nat. Rev. Immunol..

[80] Tannahill G.M., Curtis A.M., Adamik J., Palsson-McDermott E.M., McGettrick A.F., Goel G. (2013). Succinate is an inflammatory signal that induces IL-1β through HIF-1α. Nature.

[81] Chou W.C., Rampanelli E., Li X., Ting J.P.Y. (2022). Impact of intracellular innate immune receptors on immunometabolism. Cell Mol. Immunol..

[82] Cheng N., Watkins-Schulz R., Junkins R.D., David C.N., Johnson B.M., Montgomery S.A. (2018). A nanoparticle-incorporated STING activator enhances antitumor immunity in PD-L1–insensitive models of triple-negative breast cancer. JCI insight.

[83] Downey C.M., Aghaei M., Schwendener R.A., Jirik F.R. (2014). DMXAA causes tumor site-specific vascular disruption in murine non-small cell lung cancer, and like the endogenous non-canonical cyclic dinucleotide STING agonist, 2′ 3′-cGAMP, induces M2 macrophage repolarization. PLoS One.

[84] Lennarz W.J., Lane M.D. (2013).

[85] Casalino-Matsuda S.M., Wang N., Ruhoff P.T., Matsuda H., Nlend M.C., Nair A. (2018). Hypercapnia alters expression of immune response, nucleosome assembly and lipid metabolism genes in differentiated human bronchial epithelial cells. Sci. Rep..

[86] Ma Y., Nenkov M., Chen Y., Press A.T., Kaemmerer E., Gassler N. (2021). Fatty acid metabolism and acyl-CoA synthetases in the liver-gut axis. World J. Hepatol..

[87] Shortall K., Djeghader A., Magner E., Soulimane T. (2021). Insights into aldehyde dehydrogenase enzymes: a structural perspective. Front. Mol. Biosci..

[88] Itel F., Al-Samir S., Öberg F., Chami M., Kumar M., Supuran C.T. (2012). CO2 permeability of cell membranes is regulated by membrane cholesterol and protein gas channels. FASEB J..

[89] Bolshette N., Ezagouri S., Dandavate V., Karavaeva I., Golik M., Wang H. (2023). Carbon dioxide regulates cholesterol levels through SREBP2. PLoS Biol..

[90] Li Q., Hoppe T. (2023). Role of amino acid metabolism in mitochondrial homeostasis. Front. Cell Dev. Biol..

[91] Clarke C., Xiao R., Place E., Zhang Z., Sondheimer N., Bennett M. (2013). Mitochondrial respiratory chain disease discrimination by retrospective cohort analysis of blood metabolites. Mol. Genet. Metab..

[92] Phelan D.E., Reddan B., Shigemura M., Sznajder J.I., Crean D., Cummins E.P. (2024). Orphan nuclear receptor family 4A (NR4A) members NR4A2 and NR4A3 Selectively modulate elements of the monocyte response to buffered hypercapnia. Int. J. Mol. Sci..

[93] Oudaert I., Satilmis H., Vlummens P., De Brouwer W., Maes A., Hose D. (2022). Pyrroline-5-Carboxylate Reductase 1: a novel target for sensitizing multiple myeloma cells to bortezomib by inhibition of PRAS40-mediated protein synthesis. J. Exp. Clin. Cancer Res..

[94] Phang J.M., Donald S.P., Pandhare J., Liu Y. (2008). The metabolism of proline, a stress substrate, modulates carcinogenic pathways. Amino acids.

[95] Li H., Ma L., Li W., Zheng B., Wang J., Chen S. (2022). Proline metabolism reprogramming of trained macrophages induced by early respiratory infection combined with allergen sensitization contributes to development of allergic asthma in childhood of mice. Front. Immunol..

[96] Hu C.J., Wang L.Y., Chodosh L.A., Keith B., Simon M.C. (2003). Differential roles of hypoxia-inducible factor 1alpha (HIF-1alpha) and HIF-2alpha in hypoxic gene regulation. Mol. Cell. Biol..

[97] Henderson A.R. (1969). Biochemistry of hypoxia: current concepts I: an introduction to biochemical pathways and their control. Br. J. Anaesth..

[98] Mattevi A., Bolognesi M., Valentini G. (1996). The allosteric regulation of pyruvate kinase. FEBS Lett..

[99] Kierans S.J., Taylor C.T. (2021). Regulation of glycolysis by the hypoxia-inducible factor (HIF): implications for cellular physiology. J. Physiol..

[100] Iyer N.V., Kotch L.E., Agani F., Leung S.W., Laughner E., Wenger R.H. (1998). Cellular and developmental control of O2 homeostasis by hypoxia-inducible factor 1α. Genes Dev..

[101] Grimm F., Asuaje A., Jain A., Silva dos Santos M., Kleinjung J., Nunes P.M. (2024). Metabolic priming by multiple enzyme systems supports glycolysis, HIF1α stabilisation, and human cancer cell survival in early hypoxia. EMBO J..

[102] Kierans S.J., Fagundes R.R., Malkov M.I., Sparkes R., Dillon E.T., Smolenski A. (2023). Hypoxia induces a glycolytic complex in intestinal epithelial cells independent of HIF-1-driven glycolytic gene expression. Proc. Natl. Acad. Sci. U. S. A..

[103] Lin D., Yan K., Chen L., Chen J., Xu J., Xie Z. (2023). Hypoxia-induced reprogramming of glucose-dependent metabolic pathways maintains the stemness of human bone marrow-derived endothelial progenitor cells. Sci. Rep..

[104] Kuczler M.D., Olseen A.M., Pienta K.J., Amend S.R. (2021). ROS-induced cell cycle arrest as a mechanism of resistance in polyaneuploid cancer cells (PACCs). Prog. Biophys. Mol. Biol..

[105] Tello D., Balsa E., Acosta-Iborra B., Fuertes-Yebra E., Elorza A., Ordóñez Á. (2011). Induction of the mitochondrial NDUFA4L2 protein by HIF-1α decreases oxygen consumption by inhibiting Complex I activity. Cell Metab..

[106] Chandel N., Budinger G.R., Kemp R.A., Schumacker P.T. (1995). Inhibition of cytochrome-c oxidase activity during prolonged hypoxia. Am. J. Physiol..

[107] Infantino V., Santarsiero A., Convertini P., Todisco S., Iacobazzi V. (2021). Cancer cell metabolism in hypoxia: role of HIF-1 as key regulator and therapeutic target. Int. J. Mol. Sci..

[108] Wang S., Tan J., Miao Y., Zhang Q. (2022). Mitochondrial dynamics, mitophagy, and mitochondria–endoplasmic reticulum contact sites crosstalk under hypoxia. Front. Cell Dev. Biol..

[109] Pang Y., Zhu Z., Wen Z., Lu J., Lin H., Tang M. (2021). HIGD-1B inhibits hypoxia-induced mitochondrial fragmentation by regulating OPA1 cleavage in cardiomyocytes. Mol. Med. Rep..

[110] Youle R.J., Van Der Bliek A.M. (2012). Mitochondrial fission, fusion, and stress. Science.

[111] Kuo C.W., Tsai M.H., Lin T.K., Tiao M.M., Wang P.W., Chuang J.H. (2017). mtDNA as a mediator for expression of hypoxia-inducible factor 1α and ROS in hypoxic neuroblastoma cells. Int. J. Mol. Sci..

[112] Metallo C.M., Gameiro P.A., Bell E.L., Mattaini K.R., Yang J., Hiller K. (2012). Reductive glutamine metabolism by IDH1 mediates lipogenesis under hypoxia. Nature.

[113] Wang H., Airola M.V., Reue K. (2017). How lipid droplets “TAG” along: glycerolipid synthetic enzymes and lipid storage. Biochim. Biophys. Acta Mol. Cell Biol. Lipids.

[114] Ackerman D., Tumanov S., Qiu B., Michalopoulou E., Spata M., Azzam A. (2018). Triglycerides promote lipid homeostasis during hypoxic stress by balancing fatty acid saturation. Cell Rep..

[115] Bensaad K., Favaro E., Lewis C.A., Peck B., Lord S., Collins J.M. (2014). Fatty acid uptake and lipid storage induced by HIF-1α contribute to cell growth and survival after hypoxia-reoxygenation. Cell Rep..

[116] Krishnan J., Suter M., Windak R., Krebs T., Felley A., Montessuit C. (2009). Activation of a HIF1α-PPARγ axis underlies the integration of glycolytic and lipid anabolic pathways in pathologic cardiac hypertrophy. Cell Metab..

[117] Castellano J., Aledo R., Sendra J., Costales P., Juan-Babot O., Badimon L. (2011). Hypoxia stimulates low-density lipoprotein receptor–related protein-1 expression through hypoxia-inducible factor-1α in human vascular smooth muscle cells. Arterioscler. Thromb. Vasc. Biol..

[118] Perman J.C., Boström P., Lindbom M., Lidberg U., StÅhlman M., Hägg D. (2011). The VLDL receptor promotes lipotoxicity and increases mortality in mice following an acute myocardial infarction. J. Clin. Invest..

[119] Crismaru I., Pantea Stoian A., Bratu O.G., Gaman M.A., Stanescu A.M.A., Bacalbasa N. (2020). Low-density lipoprotein cholesterol lowering treatment: the current approach. Lipids Health Dis..

[120] Jung E., Kong S.Y., Ro Y.S., Ryu H.H., Shin S.D. (2022). Serum cholesterol levels and risk of cardiovascular death: a systematic review and a dose-response meta-analysis of prospective cohort studies. Int. J. Environ. Res. Public Health.

[121] Triantafyllou E.A., Georgatsou E., Mylonis I., Simos G., Paraskeva E. (2018). Expression of AGPAT2, an enzyme involved in the glycerophospholipid/triacylglycerol biosynthesis pathway, is directly regulated by HIF-1, and promotes survival and etoposide resistance of cancer cells under hypoxia. Biochim. Biophys. Acta Mol. Cell Biol. Lipids.

[122] Mylonis I., Simos G., Paraskeva E. (2019). Hypoxia-inducible factors and the regulation of lipid metabolism. Cells.

[123] Sluimer J.C., Gasc J.M., van Wanroij J.L., Kisters N., Groeneweg M., Sollewijn Gelpke M.D. (2008). Hypoxia, hypoxia-inducible transcription factor, and macrophages in human atherosclerotic plaques are correlated with intraplaque angiogenesis. J. Am. Coll. Cardiol..

[124] Rey S., Semenza G.L. (2010). Hypoxia-inducible factor-1-dependent mechanisms of vascularization and vascular remodelling. Cardiovasc. Res..

[125] Guerrini V., Gennaro M.L. (2019). Foam cells: one size doesn’t fit all. Trends Immunol..

[126] Chen L., Cui H. (2015). Targeting glutamine induces apoptosis: a cancer therapy approach. Int. J. Mol. Sci..

[127] Qing G., Li B., Vu A., Skuli N., Walton Z.E., Liu X. (2012). ATF4 regulates MYC-mediated neuroblastoma cell death upon glutamine deprivation. Cancer cell.

[128] Yoo H.C., Yu Y.C., Sung Y., Han J.M. (2020). Glutamine reliance in cell metabolism. Exp. Mol. Med..

[129] Xiang L., Mou J, Shao B., Wei Y., Liang H., Takano N. (2019). Glutaminase 1 expression in colorectal cancer cells is induced by hypoxia and required for tumor growth, invasion, and metastatic colonization. Cell. Death Dis..

[130] Le A., Lane A.N., Hamaker M., Bose S., Gouw A., Barbi J. (2012). Glucose-independent glutamine metabolism via TCA cycling for proliferation and survival in B cells. Cell Metab..

[131] Tang K., Yu Y., Zhu L., Xu P., Chen J., Ma J. (2019). Hypoxia-reprogrammed tricarboxylic acid cycle promotes the growth of human breast tumorigenic cells. Oncogene.

[132] Wise D.R., Ward P.S., Shay J.E., Cross J.R., Gruber J.J., Sachdeva U.M. (2011). Hypoxia promotes isocitrate dehydrogenase-dependent carboxylation of α-ketoglutarate to citrate to support cell growth and viability. Proc. Natl. Acad. Sci. U. S. A..

[133] Kwon D.H., Cha H.J., Lee H., Hong S.H., Park C., Park S.H. (2019). Protective effect of glutathione against oxidative stress-induced cytotoxicity in RAW 264.7 macrophages through activating the nuclear factor erythroid 2-related factor-2/heme oxygenase-1 pathway. Antioxidants.

[134] Stegen S., Van Gastel N., Eelen G., Ghesquière B., D’Anna F., Thienpont B. (2016). HIF-1α promotes glutamine-mediated redox homeostasis and glycogen-dependent bioenergetics to support postimplantation bone cell survival. Cell Metab..

[135] Xiang L., Xie G., Liu C., Zhou J., Chen J., Yu S. (2013). Knock-down of glutaminase 2 expression decreases glutathione, NADH, and sensitizes cervical cancer to ionizing radiation. Biochim. Biophys. Acta Mol. Cell Res..

[136] Lu H., Samanta D., Xiang L., Zhang H., Hu H., Chen I. (2015). Chemotherapy triggers HIF-1–dependent glutathione synthesis and copper chelation that induces the breast cancer stem cell phenotype. Proc. Natl. Acad. Sci. U. S. A..

[137] Meléndez-Rodríguez F., Urrutia A.A., Lorendeau D., Rinaldi G., Roche O., Böğürcü-Seidel N. (2019). HIF1α suppresses tumor cell proliferation through inhibition of aspartate biosynthesis. Cell Rep..

[138] Luengo A., Li Z., Gui D.Y., Sullivan L.B., Zagorulya M., Do B.T. (2021). Increased demand for NAD+ relative to ATP drives aerobic glycolysis. Mol. Cell.

[139] Westbrook R.L., Bridges E., Roberts J., Escribano-Gonzalez C., Eales K.L., Vettore L.A. (2022). Proline synthesis through PYCR1 is required to support cancer cell proliferation and survival in oxygen-limiting conditions. Cell Rep..

[140] Zhang B., Chen Y., Shi X., Zhou M., Bao L., Hatanpaa K.J. (2021). Regulation of branched-chain amino acid metabolism by hypoxia-inducible factor in glioblastoma. Cell Mol. Life Sci..

[141] To M., Yamamura S., Akashi K., Charron C.E., Haruki K., Barnes P.J. (2012). Defect of adaptation to hypoxia in patients with COPD due to reduction of histone deacetylase 7. Chest.

[142] Mirabile V.S., Shebl E., Sankari A., Burns B. (2023).

[143] Muradian K., Fraifeld V. (2021). Hypercapnia-inducible factor: a hypothesis. Ageing Longev..

[144] Park T.J., Reznick J., Peterson B.L., Blass G., Omerbašić D., Bennett N.C. (2017). Fructose-driven glycolysis supports anoxia resistance in the naked mole-rat. Science.

[145] Zions M., Meehan E.F., Kress M.E., Thevalingam D., Jenkins E.C., Kaila K. (2020). Nest carbon dioxide masks GABA-dependent seizure susceptibility in the naked mole-rat. Curr. Biol..

[146] Tolstun D.A., Knyazer A., Tushynska T.V., Dubiley T.A., Bezrukov V.V., Fraifeld V.E. (2020). Metabolic remodelling of mice by hypoxic-hypercapnic environment: imitating the naked mole-rat. Biogerontology.

